# Dominance and Epistasis Interactions Revealed as Important Variants for Leaf Traits of Maize NAM Population

**DOI:** 10.3389/fpls.2018.00627

**Published:** 2018-06-18

**Authors:** Md. M. Monir, Jun Zhu

**Affiliations:** Department of Agronomy, College of Agriculture and Biotechnology, Zhejiang University, Hangzhou, China

**Keywords:** GWAS, maize leaf traits, dominance, epistasis, genomic prediction

## Abstract

Leaf orientation traits of maize (*Zea mays*) are complex traits controlling by multiple loci with additive, dominance, epistasis, and environmental interaction effects. In this study, an attempt was made for identifying the causal loci, and estimating the additive, non-additive, environmental specific genetic effects underpinning leaf traits (leaf length, leaf width, and upper leaf angle) of maize NAM population. Leaf traits were analyzed by using full genetic model and additive model of multiple loci. Analysis with full genetic model identified 38∼47 highly significant loci (-log_10_*P_EW_* > 5), while estimated total heritability were 64.32∼79.06% with large contributions due to dominance and dominance related epistasis effects (16.00∼56.91%). Analysis with additive model obtained smaller total heritability (${\text{h}}_{\text{T}}^{2}$ ≙ 18.68∼29.56%) and detected fewer loci (30∼36) as compared to the full genetic model. There were 12 pleiotropic loci identified for the three leaf traits: eight loci for leaf length and leaf width, and four loci for leaf length and leaf angle. Optimal genotype combinations of superior line (SL) and superior hybrid (SH) were predicted for each of the traits under four different environments based on estimated genotypic effects to facilitate maker-assisted selection for the leaf traits.

## Introduction

Maize is one of the most economically important crops, source of around 24% of the total cereal production and of food, fuel, feed, or fiber in the world (Gore et al., 2009). Over the last century, grain yield of maize have increased eightfold by densely cultivating the hybrids, that can increase yields by 15∼60% relative to inbred parents (Schnable et al., 2009). Due to the economical importance, several studies have been conducted to dissect the genetic architectures of maize traits to assist in breeding programs for crop improvement (Burton et al., 2014; Lemmon and Doebley, 2014; Foerster et al., 2015). Leaf orientation traits including upper leaf angle, leaf length, and leaf width are important plant traits related with the grains of maize (Tian et al., 2011), therefore genetic dissection of these traits could assist maize breeding programs. Previously, Tian et al. (2011) identified several significant quantitative trait loci (QTLs) and SNPs for the leaf traits using QTL mapping and genome-wide association studies (GWAS). GWAS has advantages as compared to conventional linkage mapping for dissecting genetic architecture of plant complex traits (Shi et al., 2017).

In plants, the heterozygous first filial (*F*_1_) generation generally performs better than its homozygous parents, due to hybrid vigor (Shull, 1908; Huang et al., 2015). A number of QTL mapping studies demonstrated that dominance and epistasis interactions are important factors of phenotypic variations in hybrid vigor (Guo et al., 2014). Recently, GWAS for hybrid rice varieties revealed that the accumulation of numerous rear superior alleles with positive dominance is an important contributor to the heterotic phenomena (Huang et al., 2015). Other evidences have also indicated that the complexity of the genetic architecture can largely be attributed to epistasis, which plays significant roles in heteroses, inbreeding depression, adaptation, reproductive isolation, and speciation (Yang and Zhu, 2005).

With the advantages of next generation sequencing technologies, genotyping high density SNPs data across the whole genome is now possible, which might facilitate to have more information for breeding programs. Within whole genome SNPs data, a small amount of heterozygous genotypes could be found in inbred lines of different crops and animals. Most of the GWASs approaches are based on single-locus additive model ignoring non-additive effects; therefore, a study that attempt to investigate the role of heterozygous genotypes needs a statistical approach that can account for the non-additive effects. To investigate the impacts of heterozygous genotypes on leaf traits of maize NAM population, we used the full model approach with additive, dominance, epistasis, and their environmental interactions to dissect genetic architecture of complex traits. Full model approach can provide unbiased estimates of genetic parameters (Monir and Zhu, 2017b). This approach was previously used for analyzing several crop traits: Yield-related traits of rapeseed (Luo et al., 2017); Days-to-Silk of Maize NAM population (Monir and Zhu, 2017a). We also analyzed multi-loci additive model and compared the results with the full model in terms of estimated heritability and scope of breeding improvements. We estimated the breeding values for the current best line (BL), the predicted potential superior line (SL), and superior hybrid (SH), which could help breeders for further trait manipulation.

## Materials and Methods

### Genotype and Phenotype Data

We used the maize NAM population with 4,892 lines from 25 families, which were constructed by crossing 25 diverse inbred lines with B73, and then self-pollinating for five generations (Gore et al., 2009; Tian et al., 2011). Three leaf traits (leaf length, leaf width, and upper leaf angle) were scored in nine trials during the summer and winter of 2006 and 2007 in six locations: Aurora, New York, Urbana, Illinois, Clayton, and North Carolina, United States, in 2006 and 2007; Columbia, Missouri, Homestead, Florida, United States and Ponce, Puerto Rico in 2006 (Tian et al., 2011). We downloaded the genotype and phenotype data sets from the website https://www.panzea.org/. In this study, we analyzed the data collected from four locations of United States (Aurora, Urbana, Columbia, and Homestead, United States) in 2006. It was better to include data collected from Clayton in 2006 for analysis. However, currently it is quite impossible for analyzing such a large data set (total number of observations in five environment is ∼ 25,000). The total phenotypic observations used for association analyses were 12,241, 17,613, and 17,916 for leaf angle, leaf width, and leaf length.

### Statistical Analysis

Two distinct approaches were used for GWAS of traits: generalized multi-factor dimensionality reduction (GMDR) method to scan SNPs by 1D for main effects, 2D and 3D for epistasis interactions using module *GMDR*-GPU (Zhu et al., 2013) of *QTXNetwork*, and then association mapping was conducted on detected SNPs by using *QTS* module of *QTXNetwork.* We used two different models (full model and additive model) for association mapping (Monir and Zhu, 2017b): The full model includes fixed loci effects (*a, d, aa, ad, da, dd*), and random effects of loci by environment interaction effects (*ae, de, aae, ade, dae, dde*); the additive model includes fixed additive effects (*a*), and random additive by environment interaction effects (*ae*).

The mixed linear model framework with Henderson method III (Searle et al., 2009) was used to construct the *F*-statistic test for association analyses. Permutation test was conducted by a total of 2,000 times for calculating the critical *F*-value to control the experiment-wise type I error (α_EW_ < 0.05). The genetic effects of quantitative trait SNPs (QTSs) were estimated by using the MCMC (Markov Chain Monte Carlo) algorithm with 20,000 Gibbs sample iterations (Yang et al., 2007, 2008; Qi et al., 2014; Locke et al., 2015); and the critical experiment-wise *P*_EW_-value for genetic effects was calculated by controlling the experiment-wise type I error (*P*_EW_ < 0.05).

### Annotation of Genes Related With QTSs

Candidate genes of detected QTSs were inferred from Gramene database^1^. We searched functions of candidate genes in Uniprot with the accession number of the genes collected from Gramene database. In addition, we carried out the literature survey to know the functions of candidate genes by using NCBI-Pubmed and others biological database like GeneCards.

## Results

### Estimated Heritability for Leaf Traits

We estimated heritability of quantitative trait SNPs (QTSs) by full model approach for upper leaf angle (ULA), leaf width (LW), and leaf length (LL) of maize lines. Estimated heritability by using full model was 64.32% for ULA, 79.06% for LW, and 76.77% for LL. Large portion of the estimated total heritability was due to dominance and dominance related epistasis interaction for leaf length $\left(h_{D+}^{2}\hat{=}56.91\%\right)$ and leaf width $\left(h_{D+}^{2}\hat{=}55.20\%\right)$ (**Table 1**). Total dominance related heritability was smaller for ULA $\left(h_{D+}^{2}\hat{=}16.00\%\right)$; there was only one QTS with highly significant dominance effect (Supplementary Table S1). Large portion of the phenotypic variation of ULA was due to additive and additive × additive epistasis $\left({\text{h}}_{\text{A}}^{2}+{\text{h}}_{\text{AA}}^{2}\hat{=}42.09\%\right)$, suggesting the phenotypic variations of upper leaf angle were mostly controlled by the additive and additive × additive epistasis effects of multiple loci. Genetic properties of LW and LL are different from ULA for NAM population. Environmental specific heritability $\left({\text{h}}_{\text{GE}}^{2}\right)$ was relatively small for the leaf traits (7.32% for ULA, 5.48% for LW, and 4.98% for LL).

**Table 1:** Estimated heritability (%) of genetic effects in full model for three leaf traits of maize.

Trait	${\text{h}}_{\text{A}}^{2}$	${\text{h}}_{\text{D}}^{2}$	${\text{h}}_{\text{AA}}^{2}$	${\text{h}}_{\text{AD}}^{2}$	${\text{h}}_{\text{DA}}^{2}$	${\text{h}}_{\text{DD}}^{2}$	${\text{h}}_{\text{AE}}^{2}$	${\text{h}}_{\text{DE}}^{2}$	${\text{h}}_{\text{AAE}}^{2}$	${\text{h}}_{\text{T}}^{2}$	${\text{h}}_{\text{D+}}^{2}$
Angle	34.60	11.15	7.49	2.17	1.59	0.00	6.23	1.09	0.00	64.32	16.00
Width	13.40	10.93	3.55	4.15	16.13	25.42	4.52	0.28	0.68	79.06	56.91
Length	12.35	8.21	7.31	7.66	5.62	30.64	0.96	3.07	0.95	76.77	55.20

*${\text{h}}_{\text{A}}^{2}$ = heritability of additive effects; ${\text{h}}_{\text{D}}^{2}$ = heritability of dominance effects; ${\text{h}}_{\text{AA}}^{2}$ = heritability of additive × additive epistasis; ${\text{h}}_{\text{AD}}^{2}$ = heritability of additive × dominance epistasis; ${\text{h}}_{\text{DA}}^{2}$ = heritability of dominance × additive epistasis; ${\text{h}}_{\text{DD}}^{2}$ = heritability of dominance × dominance epistasis; ${\text{h}}_{\text{AE}}^{2}$ = heritability of additive by environmental interaction effects; ${\text{h}}_{\text{DE}}^{2}$ = heritability of dominance by environmental interaction effects; ${\text{h}}_{\text{AAE}}^{2}$ = heritability of additive × additive epistasis by environmental interaction effects; ${\text{h}}_{\text{T}}^{2}$ = total heritability; ${\text{h}}_{\text{D+}}^{2}$ = sum of dominance related heritability.*

### Genetic Effects of Detected Loci for Three Leaf Traits Using Full Model Approach

#### Upper Leaf Angle

**FIGURE 1:**
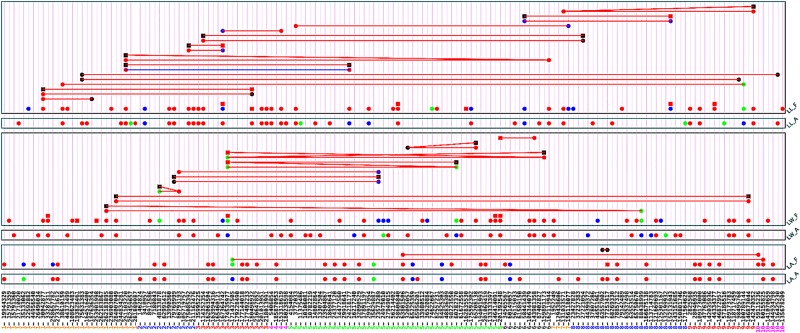
G×G plot of detected significant QTSs (*P*_EW_ < 0.05) for three traits by using full model and additive model approaches. Circle: QTS with additive effect; square: QTS with dominant effect; line between two QTSs: epistasis effect; red color: QTS with general effects for two environments; green color: QTS with environment-specific effects; blue color: QTS with both general and environment-specific effects; black color: QTS with significant epistasis effects but without detected individual effects.

Upper leaf angle is an important trait of leaf architecture for efficient light capture and high dense planting. Association analysis by using full model identified 38 QTSs with experimental-wise highly significant effects (-log_10_*P_EW_* > 5) for upper leaf angle, 37 QTSs with individual genetic effects and three pairs of QTSs with epistasis interactions (**Figure 1** and Supplementary Table S1). Among the highly significantly identified QTSs, 34 QTSs had additive effects (positive for 19 QTSs and negative for 15 QTSs), 2 QTSs had only environmental specific additive effects, and 1 QTS had only dominance effect. In our analysis, we coded the non-B73 homozygous genotypes (*QQ*) by 1 and the B73 homozygous genotypes (*qq*) by -1.

We identified several QTSs with extremely highly significant effects. For example, QTS S1_35133062, the variant of unknown protein coding gene *GRMZM2G136513* was identified with most highly significant positive additive effect (a ≙ 0.841, -log_10_*P_EW_* = 99.7), that could explain 2.82% of phenotypic variations for increasing upper leaf angle by non-B73 alleles but decreasing upper leaf angle by B73 alleles (Supplementary Table S1). This QTS also had significant additive effects positive in environment 2 (ae_2_ ≙ 0.389, -log_10_*P_EW_* = 6.7) but negative in environment 3 (ae_3_ ≙ -0.376, -log_10_*P_EW_* = 6.4). QTS S5_84825303 had significant positive additive effect (a ≙ 0.781, -log_10_*P_EW_* = 83.2) and additive × environmental interactions (a + ae_1_ ≙ 1.312, a + ae_2_ ≙ 0.324). Again, several genes corresponding to detected QTSs had relevant functions to leaf architectures. For example, QTS S5_84825303 near gene (*GRMZM2G382914*) has influences on the three phases to the light-independent reactions, collectively called the Calvin cycle: carbon fixation, reduction reactions, and ribulose 1,5-bisphosphate (RuBP) regeneration (Khan et al., 2010). The Calvin cycle occurs only when light is available and plants do not carry out the process during night-time. QTS S3_216647852, near to protein coding gene *GRMZM2G464976* regulating maize leaf growth (Candaele et al., 2014), had highly significant positive additive effect (a ≙ 0.712, -log_10_*P_EW_* = 72.4). Moreover, QTS S5_63801506, near to DNA-binding protein MNB1B (*GRMZM5G834758*), had highly significant additive effect (a ≙ 0.549, -log_10_*P_EW_* = 43.0). The gene *GRMZM5G834758* including the High Mobility Group Box (HMG box) gene family, and proteins of the gene family are involved in germination, seeding growth and stress response (Lildballe et al., 2008; Candaele et al., 2014). The gene *GRMZM5G834758* showed highly expressed through the entire 192-*h* time course in recent experimental study, suggesting that it might regulate multiple processes (Yu et al., 2015). QTS S5_194091993, the variants of HMGc1 protein gene (*GRMZM2G013821*) had negative additive effect (a ≙ -0.598, -log_10_*P_EW_* = 50.6). HMG-box domains are found in HMG proteins, which are involved in the regulation of DNA-dependent processes such as transcription, replication, and DNA repair, all of which require changing the conformation of chromatin (Thomas, 2001). Most of the genes corresponding to the identified QTSs for upper leaf angle are uncharacterized protein (24 uncharacterized protein-coding genes and 14 known genes) (Supplementary Table S1).

Three pairs of epistasis were identified using the full model, in which none of them had highly significant dominance related epistasis effects. The most significant and largest additive × additive (*aa*) epistasis effect was estimated between the QTSs S8_63557902 and S8_77253417 (aa ≙ 0.722, -log_10_*P_EW_* = 72.2, ${\text{h}}_{\text{aa}}^{2}$ ≙ 4.15%). Hence, additive genetic effects of many loci with small effects and additive × additive (*aa*) epistasis interactions are the major genetic effects for controlling upper leaf angle of maize NAM population.

#### Leaf Width

Leaf width is another important trait of leaf architecture. Analysis with full model approach identified total 47 QTSs with experimental-wise highly significant effects (-log_10_*P_EW_* > 5) on the leaf width: 45 QTSs with individual genetic effects (39 QTSs with additive effects, 2 QTSs with only dominance effect, 4 QTSs with both additive and dominance effects), and 9 pairs of epistasis interactions (**Figure 1** and Supplementary Table S2). Full model approach estimated large amount of heritability due to dominance and dominance related epistasis effects (${\text{h}}_{\text{D+}}^{2}$ ≙ 55.20%) for leaf width. Individual genetic effects of the QTSs were relatively small as compared to the epistasis interactions, and dominance related epistasis interactions were identified with large effect. Ratios of heterozygote genotypes were 2.69∼7.17% for the loci, which were identified with highly significant dominance effects. And, the ratios of heterozygote genotypes were 3.30∼8.97% for the loci, which had dominance related epistasis effects. Therefore, only a small portion of individuals had heterozygote genotypes with large impacts of dominance and dominance related epistasis effects. It was illustrated for importance of analyzing heterozygous genotypes in GWAS even if for inbred lines. We analyzed 17,613 individual observations that were scored under four different trials, and around 474∼1,580 individuals had heterozygote genotypes across the loci. It refers that a very small portion of heterozygotes in SNPs of NAM population could result in large proportion of dominance and dominance related epistasis interactions.

QTS S8_125301167, the variant of ZIP (zinc and iron regulated transporter protein) metal ion transporter family gene *GRMZM2G034551*, had most highly significant additive effect (a ≙ 1.578, -log_10_*P_EW_* = 93.5). However, additive effect of this QTS largely varied across environments, maximum in environment 1 (a + ae_1_ ≙ 3.682) and minimum in environment 3 (a + ae_3_ ≙ -0.076). Influences of the metal transporter protein in plants might depend on environment, especially on soil and weather where the plants were grown. QTS S6_106344079, the near variant of BCR family protein gene *GRMZM2G071638*, had second most highly significant additive effect (a ≙ 1.409, -log_10_*P_EW_* = 73.8). QTS S6_143981358, the near variant of protein gene *GRMZM2G424582* was identified with high significance and large positive additive effect (a ≙ 1.369, -log_10_*P_EW_* = 69.0), whereas the gene has relationship with maize seed developmental traits (Walley et al., 2013). The largest negative additive effect (a ≙ -1.289, -log_10_*P_EW_* = 61.2) was estimated for QTS S5_33980512, which is the variant of uncharacterized protein-coding gene *GRMZM2G075150*. Two QTSs (S1_193828461 and S1_253901998) had only highly significant dominance effects. QTS S3_174535977, the variant of protein gene *GRMZM2G129428*, had highly significant large dominance (d ≙ -3.511, -log_10_*P_EW_* = 16.4) and environment specific additive effects, whereas dominance effect of the QTS could explain 2.59% phenotypic variations for decreasing leaf width by heterozygous genotypes. Gene ontology showed two molecular functions of *GRMZM2G129428*: metal ion binding and nucleic acid binding^2^. Among the 9 highly significant epistasis pairs, 8 pairs had dominance related epistasis effects. Epistasis of S2_86793193 × S5_23573289 was identified with highly significant additive × additive epistasis effect (aa ≙ 0.777, -log_10_*P_EW_* = 21.3), which are the near variant of genes *GRMZM2G052268* (F-box domain containing protein) and *GRMZM2G074543* (*Yabby9* protein), respectively. F-box domain containing proteins are associated with cellular functions such as signal transduction and regulation of the cell cycle (Craig and Tyers, 1999). Maize *YABBY* gene family members *Yabby9* and *Yabby14* were expressed in the adaxial domain of leaves, play a role in lateral leaf outgrowth (Juarez et al., 2004; Dai et al., 2007). Large dominance × dominance epistasis interactions were identified between the variants of *Yabby9* and *GRMZM2G102699* genes (dd ≙ 5.078, -log_10_*P_EW_* = 11.5); and between the variants of *GRMZM2G129428* and *GRMZM2G108775* genes (dd ≙ 7.562, -log_10_*P_EW_* = 20.1). *GRMZM2G108775* is FMN-linked oxidoreductases superfamily protein gene. Highly significant additive × additive (*aa*) and additive × dominance (*ad*) epistasis interactions were identified between the variants S5_32095057 of *AC233949.1_FG004* (cell division cycle protein 48) and S5_65208138 of *GRMZM2G057000* (Brassinosteroid biosynthesis-like protein). Many individual loci were identified with small effects but several pairwise epistasis interactions with large effects for leaf width of maize NAM population, whereas dominance and dominance related epistasis interactions were revealed as important contributor to phenotypic variations of the trait.

#### Leaf Length

We identified 46 QTSs with highly experimental-wise significant (-log_10_*P_EW_* > 5) effects underpinning to the leaf length using full model approach (**Figure 1** and Supplementary Table S3). Of which, 41 QTSs with individual effects and 13 pair-wise epistasis interactions were identified. Full model identified 37 QTSs with highly significant additive effects contributing around 12.22% phenotypic variation. Therefore, most portions of the estimated total genetic variations due to additive effects had highly significant effects, including negative additive effects of 15 QTSs (${\text{h}}_{\text{A-}}^{2}$ ≙ 4.80%), and positive additive effects of 22 QTSs (${\text{h}}_{\text{A+}}^{2}$ ≙ 7.42%). Therefore, QTSs with positive additive effects contributed more than QTSs with negative additive effects for leaf length of maize NAM population. QTS S6_162119944 had most significant and largest negative additive effects (a ≙ -11.479, -log_10_*P_EW_* = 93.1) that contributed around 1.04% of the phenotypic variation. This QTS is the near variant of protein coding gene *GRMZM2G093346* (*APx1*-Cytosolic Ascorbate Peroxidase 1) involving in glutathione metabolism, ascorbate, and aldarate metabolism pathways. Cytosolic ascorbate peroxidase 1 is a central component of the reactive oxygen gene network of Arabidopsis (Davletova et al., 2005) and photosynthetic electron transport regulates the expression of the gene in Arabidopsis during excess light stress (Karpinski et al., 1997). QTS S1_38610159 had highly significant negative additive effect (a ≙ -8.513, -log_10_*P_EW_* = 54.5), whereas this QTS is near variant of the cysteine proteinases superfamily protein gene *GRMZM2G128444*. QTS S8_79142489, the near variant of the uncharacterized protein-coding gene *GRMZM2G040467*, had the largest positive additive effect (a ≙ 11.424, -log_10_*P_EW_* = 97.4) that contributed 1.03% of phenotypic variations. Association analysis identified 6 QTSs with highly significant dominance effects, whereas ratios of the heterozygote genotypes corresponding to the QTSs were only 4.35 ∼ 8.89%. Therefore, around 779∼1,593 individual plants had heterozygote genotypes across the QTSs among the total 17,916 individual observations. QTS S5_58606840, the variant of chloroplast thylakoid lumen protein coding gene *GRMZM5G854533*, was identified with highly significant additive (a ≙ 4.159, -log_10_*P_EW_* = 13.1) and dominance (d ≙ 12.308, -log_10_*P_EW_* = 10.3) effects. The thylakoid lumen provides the environment for oxygen evolution, plastocyanin-mediated electron transfer, and photoprotection; and more recently lumenal proteins have been revealed to play roles in numerous processes, most often linked with regulating thylakoid biogenesis and the activity and turnover of photosynthetic protein complexes, especially the photosystem II and NAD(P)H dehydrogenase-like complexes (Gnatiuc et al., 2013).

We identified 13 pairs of epistasis interactions, and estimated effects of the epistasis interactions were relatively large as compared to the main effects. Therefore, leaf length could be controlled by several QTSs with small main effects and large epistasis interactions. Significant additive × additive epistasis interaction effects were observed for all significant QTSs pairs, whereas dominance related epistasis effects were observed for seven pairs of QTSs. Ratios of the heterozygous genotypes were 2.90∼6.74% corresponding the QTSs that had dominance related epistasis effects. Highly significant negative additive × additive epistasis effect (aa ≙ -10.700, -log_10_*P_EW_* = 80.8) and positive dominance × dominance epistasis effect (dd ≙ 25.941, -log_10_*P_EW_* = 5.5) were identified for the QTSs S6_58441439 (*GRMZM2G103230*) × S8_152131943 (*GRMZM2G060886*). Two pairs of epistasis interactions were due to S1_30042877 (*GRMZM2G110131*) with S1_187636354 (*GRMZM2G132763*) and S3_180498932 (*GRMZM2G064853*). The gene *GRMZM2G110131* is a member of tify domain/CCT motif transcription factor protein gene family, and recent study showed that the members of the tify gene family has tissue-specific expression patterns in various maize development stages and in response to biotic and abiotic stresses (Zhang et al., 2015). Genetic architecture of leaf length was similar to genetic architecture of leaf width, whereas dominance and dominance related epistasis effects were important variants for the traits.

### Pleiotropic Loci of the Leaf Traits

We tabulated the QTSs that were commonly identified for more than one leaf trait and observed that none of the QTSs associate with all of the three leaf traits (**Table 2**). Eight QTSs associated with both leaf length and leaf width; four QTSs associated with both upper leaf angle and leaf length; however, there were no pleiotropic loci associated with both leaf width and upper leaf angle.

**Table 2:** Identified pleiotropic loci for the leaf traits.

QTS	Gene	Trait	Gene descriptions
S1_30042877	GRMZM2G110131	Length, width	PnFL-2; Putative tify domain/CCT motif transcription factor family protein
S1_44373499	GRMZM2G107867	Length, width	SNF1-related protein kinase
S1_108623483	GRMZM2G036567	Length, width	Uncharacterized protein
S2_79769999	GRMZM2G102699	Length, width	Uncharacterized protein
S2_211163476	GRMZM2G021567	Length, width	Pentatricopeptide repeat (PPR) superfamily protein
S3_167364742	GRMZM2G093119	Length, width	Uncharacterized protein
S4_153318619	GRMZM2G463471	Length, width	Actin-depolymerizing factor
S5_133333397	GRMZM2G105494	Length, width	Uncharacterized protein
S9_142035936	GRMZM5G831481	Length, angle	Uncharacterized protein
S9_109173000	GRMZM2G103647	Length, angle	Light-inducible protein CPRF-2; Putative bZIP transcription factor superfamily protein
S10_60155825	GRMZM2G079777	Length, angle	Vacuolar ATP synthase subunit D 1
S10_144934798	GRMZM2G527256	Length, angle	Uncharacterized protein

*QTS, quantitative trait SNP; Gene, corresponding near or holder gene of loci; Trait, traits were analyzed; Gene descriptions, descriptions of the candidate genes.*

Pleiotropic QTS S1_30042877, the variants of putative TIFY domain/CCT motif transcription factor family protein gene *GRMZM2G110131*, had negative additive effect on leaf width (a ≙ -0.748, -log_10_*P_EW_* = 21.2), but positive additive effect on leaf length (a ≙ 4.783, -log_10_*P_EW_* = 17.4). Four QTSs (S1_44373499, S2_211163476, S3_167364742, and S5_133333397) had positive additive effect on both leaf length and leaf width. QTS S1_44373499 is the variant of SNF1-related protein kinase gene *GRMZM2G107867* that play an role in multitude of cellular processes, including division, proliferation, apoptosis, and differentiation (Manning et al., 2002). QTS S2_211163476 is the variant of pentatricopeptide repeat (PPR) superfamily protein gene *GRMZM2G021567*, whereas PPR proteins have been implicated in many crucial functions broadly involving organelle biogenesis and plant development (Saha et al., 2007). QTSs S2_79769999 and S4_153318619 had negative additive effect on both leaf length and width. Two pleiotropic loci (S10_60155825 and S10_144934798) for upper leaf angle and leaf length had positive additive effects for both traits. QTS S9_109173000, the variant of light-inducible protein CPRF-2 (*GRMZM2G103647*) had negative additive effect (a ≙ -0.556, -log_10_*P_EW_* = 43.9) for upper leaf angle, but positive additive effect (a ≙ 5.955, -log_10_*P_EW_* = 26.3) for leaf length. Variants of four uncharacterized protein-coding genes had significant effects on both leaf length and leaf width.

### Predicted Genotypic Values for Different Genotypes

The genetic information obtained from the association studies could be used for designing SLs and hybrids for further crop improvement. Based on the genetic effects, the total genotypic value corresponding to each individual line can be calculated. Along with the provided association mapping results, total genotypic effects of BL, SL, and SH can also be predicted. By utilizing the association mapping results, we predicted BL, SL, and SH for three leaf traits, and tabulated the overall total genetic effects and total genetic effects under four different environments for the leaf traits (**Table 3**).

**Table 3:** Prediction of total genetic effects of leaf traits using full model.

Entry	*G*	*G+GE1*	*G+GE2*	*G+GE3*	*G+GE4*
**ULA (μ ≙ 62.84)**					
*QQ*	5.86	5.47	6.05	5.87	6.94
*qq*	-2.60	-2.21	-2.79	-2.61	-3.68
*F*_1_	4.77	3.96	5.43	4.77	4.77
Best line (+)	15.22	16.80	15.83	13.93	15.46
Superior line (+)	16.61	18.26	17.25	16.09	18.54
Superior hybrid (+)	19.31	21.22	20.37	18.79	20.81
**LW (μ ≙ 85.97)**					
*QQ*	3.51	5.70	3.97	2.89	5.75
*qq*	-2.09	-4.28	-2.55	-1.46	-4.32
*F*_1_	5.97	5.97	5.97	4.96	7.26
Best line (-)	-23.22	-26.19	-24.28	-21.60	-23.41
Superior line (-)	-26.54	-30.27	-29.33	-26.07	-28.49
Superior hybrid (-)	-44.07	-46.18	-50.49	-42.57	-42.72
**LL (μ ≙ 743.77)**					
*QQ*	56.48	62.17	56.48	40.53	64.33
*qq*	-33.17	-38.86	-33.17	-17.22	-51.87
*F*_1_	-0.26	-0.26	-0.26	-2.11	-0.26
Best line (-)	-150.17	-155.86	-150.17	-135.14	-146.68
Superior line (-)	-167.46	-213.19	-167.46	-211.02	-194.29
Superior hybrid (-)	-269.11	-312.30	-269.11	-310.12	-293.22

**QQ*, total genetic value of predicted line by only *QQ* homozygous genotypes of all significant loci; *qq*, total genetic value of predicted line by only *qq* homozygous genotypes of all significant loci; Best line (+), maximum positive genetic value of existing line; Best line (-), maximum negative genetic value of existing line; Superior line (+), predicted line with maximum positive genetic value by combining homozygous genotypes (*QQ, qq*) for all significant loci; Superior line (-), predicted line with minimum negative genetic value by combining homozygous genotypes (*QQ*, *qq*) for all significant loci; *F*_*1*_, predicted line with total genetic value only for heterozygous genotypes (*Qq*) corresponding to all significant loci; Superior hybrid (+), predicted line with maximum positive genetic value by combining homozygous (*QQ, qq*) and heterozygous (*Qq*) genotype for all significant loci; superior hybrid (-), predicted line with maximum negative genetic value by combining homozygous (*QQ, qq*) and heterozygous (*Qq*) genotype for all significant loci.*

For ULA, overall total genetic effect of the non-B73 allele homozygous (*QQ*) combinations was 5.86° across environments, but variant (5.47°∼6.94°) in four environments. Predicted total genetic effect for *F*_1_ hybrid (4.77°) was smaller than non-B73 allele homozygous (*QQ*) genotypes. Maximum total genetic effect across environments was revealed for the line Z019E0033 (15.22°) called as the BL across environments, whereas environment specific BLs were Z014E0005 (16.80°) in environment 1 (Urbana), Z021E0060 (15.83°) in environment 2 (Aurora), and Z019E0033 in two environments [(13.93° in environment 3 (Homestead) and 15.46° in environment 4 (Columbia)]. Environment specific total genetic effects were very large for the line Z014E0005 across all of the four different environments (16.80°, 15.79°, 13.38°, and 15.40° in environments 1∼4, respectively) that were close to the total genetic effects of environmental specific BLs. Total genetic effect of the BL in environment 4 (Homestead) was smaller as compared to others three locations. The predicted superior positive line [superior line (+)] could provide insight for crop improvement along with the optimum homozygous genotypes (*QQ, qq*) combinations. Total overall genetic effect of the predicted SL had large leaf angle (16.61°), which is similar to the existing BL (Z019E0033), suggesting that the less scope of further improving upper leaf angle over the BLs. Again, the total genetic effect of the SH, that exhaust the optimum combination of homozygous (*QQ, qq*) and heterozygous (*Qq*) genotypes, had 19.31° upper leaf angle which is 4.04° larger than the existing line, referring that the predicted SH has greater scope than the predicted SL for further improvement. Total genetic effects of predicted SL and SH were also smaller in environment 4 (Homestead) as compared to others three locations. Comparison between Z014E0005 and SH across the identified loci showed that genotypes of 15 QTSs were different to each other, whereas genotypes of 9 QTSs had heterozygous genotypes for SH. Z014E0005 had no heterozygous genotypes over identified loci. We tabulated optimum genotypes corresponding to loci of the predicted lines (Supplementary Table S4) that could be helpful to breeders for the further crop improvement.

For leaf width, the overall total genetic effect for B73 allele homozygous genotypes (*qq*) combination had -2.09 mm, and minimum in Columbia (-4.32 mm). Existing line Z024E0055 had revealed as the overall and environment specific BL, with its minimum value in Urbana (-26.19 mm) and maximum value in Homestead (-21.60 mm). Predicted SL had mostly similar total genetic effect as compared to the existing BL, suggesting the existing line already exhausted the optimum homozygous genotypes combination and has none or very little scope for further improvement (**Table 3**). However, predicted SH had lower overall total genetic effect (-44.07 mm), which is 20.85 mm smaller than the overall total genetic effect of the BL, suggesting that there is a great scope for further trait manipulation with the predicted SH. By comparing SH (-) with Z024E0055, we observed that the genotypes of 24 QTSs were different from each other. SH (-) require heterozygous genotypes corresponding to 9 QTSs, whereas Z024E0055 had major or minor allele homozygous genotypes corresponding to the QTSs. Moreover, SH (-) need major allele homozygous genotypes but Z024E0055 had heterozygous genotypes for 4 QTSs.

For leaf length, overall total genetic effect for B73 allele homozygous (*qq*) combinations had -33.17 mm, for non-B73 allele homozygous (*QQ*) combinations had 56.48 mm. Total genetic effect of B73 allele homozygous combinations had minimum in environment 3 (Columbia) (-51.87 mm) and maximum in environment 4 (Homestead) (-17.22 mm), the total genetic effects did not differ greatly across others 2 different locations. Predicted total genetic effect for *F*_1_ hybrid was lower than the total genetic effect of non-B73 allele homozygous (*QQ*). Minimum overall total genetic effect was revealed for line Z024E0103 (-150.169 mm) that also had lowest value in environment 1 (Aurora) and environment 2 (Urbana). Z024E0048 and Z024E0170 had minimum value in environment 3 (Columbia) and environment 4 (Homestead). Predicted superior line [SL (-)] had lower total genetic effect than the best line [BL (-)], refer the scopes to reduce leaf length using the predicted line. Predicted superior hybrid [SH (-)] had very lower total genetic effect (-220.27 mm), that was too smaller than the total genetic effect of the negative BL, suggesting great scope to reduce leaf length with the SH. By comparing SH (-) with Z024E0103, we observed that genotypes of 28 significant QTSs were different to each other.

We compared the three different BLs of leaf traits in terms of increasing upper leaf angle, but decreasing leaf width and leaf length. BL (-) for LL and LW were from 24th family, but the BL for ULA was from 14th family of NAM population. BL of ULA (Z014E0005) had average effect for width (total genetic effect was close to zero) and large genetic effect for length. However, BL **(-)** of LW had smaller total genetic effect for LL, and BL (-) of LL also had smaller total genetic effect for LW. Genotypes of 34 QTSs were different for BL of LL and LW as compared to the SH of ULA across the identified loci. BL (-) of LW and LL had mostly similar total genetic effects to its SL (-). Therefore, manipulating 34 QTSs of Z024E0103 or Z024E0055 might achieve much improvement for leaf orientation that could help in high dense cultivation as compared to the existing lines.

For further decreasing length and width, Z024E0103 or Z024E0055 require fewer loci manipulation as compared to Z014E0005. Therefore, SH for the three leaf traits could be obtained by choosing BL (-) of LL or LW for future improving the leaf traits.

### Analyses Results Using Additive Model Approach

We also used additive model approach to analyze the leaf traits and observed the substantial differences in analyses results. Since additive model approach ignores non-additive and epistasis effects, the estimated total heritability was much smaller (${\text{h}}_{\text{T}}^{2}$ ≙ 48.86% for ULA_A, 40.03% for LW_A, and 19.36% for LL_A) than the full model approach (${\text{h}}_{\text{T}}^{2}$ ≙ 64.32% for ULA, 79.06% for LW, and 79.79% for LL) due to the missing heritability problem of additive model. Therefore, ignoring dominance and epistasis interactions could have large impacts on estimating heritability of complex traits. The number of highly significant (-log_10_*P_EW_* > 5) loci was smaller than the full model (38∼47 loci in full model and 30∼36 loci in additive model). For upper leaf angle, the prediction of total genetic value of positive SL was smaller for additive model [SL (+) = 13.23° for ULA_A] than full model [SL (+) = 16.61° for ULA] (**Table 3** and Supplementary Table S5). Again for leaf width and leaf length, the predictions of total genetic value of negative superior line [SL (-) = -19.99 mm for LW_A and -131.86 mm for LL_A] were larger than the predictions by using full model [SL (-) = -26.54 mm for LW and -167.46 mm for LL]. Therefore, full model approach can provide more information for detected loci, which may create more scope for crop improvement.

## Discussion

Maize is an important economical crop in the world. With the advantages of whole genome sequencing technology, the genetic architecture of complex traits of maize inbred lines have been dissected (Tian et al., 2011) that might assist for further breeding improvement. Tian et al. (2011) analyzed three leaf traits (upper leaf angle, leaf width and length) using linkage analysis (by joint inclusive interval mapping approach) and GWAS (using sub sampling based approach) for nested association mapping (NAM) population, identified 30∼36 QTLs and 203∼295 SNPs, respectively. However, 23% of the genotypes were missing in the set of 1.6 million SNPs, which were imputed in their study based on a haplotype clustering algorithm implemented in fastPHASE version 1.3 (Tian et al., 2011). In the real data set, there were no heterozygous genotypes rather than the missing genotypes. Maize is a cross-pollinated crop with unique and separate male (tassel) and female (ear) organs, and maize breeding has unique features that are different from other self-pollinated grain crops. Heterozygous loci might be observed across the whole genome for inbred lines of different species even if after many generations of self-matting, because selections are generally conducted based on phenotypes. Small proportion of heterozygote genotypes were observed in diverse inbred lines of different organisms (e.g., mice, cotton, rice, etc.). Recent study (Liyuan, 2016) observed 7% heterozygotes in cotton diverse inbred lines, and found large contributions of dominance and dominance related epistasis effects for four yield traits. A small proportion of heterozygous genotypes are also expected over the whole genome in recombinant inbred population. Maize NAM population was generated by only five-generation self-crossing and therefore a substantial amount of heterozygotes is expected. In this analysis, we used an indicator variable for missing genotypes (1 if genotype is missing and otherwise 0) to test their effects on phenotype. In this case, effect of the missing genotypes could be significant if they have different effects from the average effects of the corresponding homozygous genotypes. This type of test is equivalent to imputing the missing genotypes of maize NAM population by heterozygous genotypes for *QTXNetwork* software. If missing genotypes corresponding to testing loci have no effects of dominance and/or dominance related epistasis, they would not be significantly identified, because standard error (SE) should be very high in that case. For justifying this hypothesis, we conducted a simple Monte-Carlo simulation by assuming that the missing genotypes are homozygotes and generated a set of phenotypic data after replacing the missing genotypes of the selected loci by homozygous genotypes (*QQ* or *qq*) randomly. However, missing genotypes were treated as heterozygote genotypes in time of analyses. In simulation we assumed that the missing genotypes are heterozygotes. If there were no dominance related effects of these loci, we still cannot identify dominance related effects. Simulation results showed that if the missing genotypes are homozygotes and we assume them as heterozygotes in the time of analysis then dominance and dominance related epistasis interaction would not be highly significant (Supplementary Table S6). This simulation provided the validity of our analysis procedure.

In real data analysis, we observed significant effects of the imputed heterozygous genotypes of the identified loci on the leaf traits. We estimated that large portion of heritability was due to dominance and dominance-related epistasis for 3 leaf traits (${\text{h}}_{\text{D+}}^{2}$ ≙ 16.00% for ULA, 56.91% for LL, and 55.20% for LW). Our identified QTSs were not exactly same as reported by Tian et al. (2011), but several loci are nearby (Supplementary Table S7). And some of the identified QTSs were within their reported QTL support interval. Again, we identified several highly significant epistasis loci. That could be due to different statistical approach or due to genotyping issue. Statistical approach of *QTXNetwork* allow to use increased number of sample size with replication of individual observations under different environments (total sample size = number of individual lines × number of environments). Previous approach (Tian et al., 2011) used best linear unbiased prediction (BLUP) of individual lines from nine different environments that ultimately reduced the sample size (total sample size = number of individual lines). Large sample size has more detection power in comparison to small sample size. Moreover, another cause of detecting epistasis effects in our approach could be genotyping issue, because we identified several highly significant dominance and dominance related epistasis effects. We identified only few pleiotropic loci for the leaf traits, whereas no pleiotropic loci were identified between upper leaf angle and leaf width.

We predicted the SHs using association analysis results and observed great scopes of further improvement by accounting the heterozygous genotypes. For example, there were 49 QTSs for controlling leaf length with total predicted genetic effect -269.11 mm for the predicted superior hybrid (-) and -167.46 mm for the predicted superior line (-). The difference was due to only heterozygote effects of 13 QTSs (S1_30042877, S1_38610159, S1_187636354, S1_251103220, S2_79769999, S3_180498932, S4_153318619, S5_162835530, S8_152131943, S9_2825568, S9_98495768, S9_100789144, S9_142470352). Therefore, manipulating these 13 heterozygote loci could further decrease leaf length up to -101.65 mm for superior hybrid (-). Total genetic effects of BLs in Homestead were smaller or larger as compared to others three locations. Geographically, three locations (Urbana, Aurora, and Columbia) are relatively near as compared to Homestead, and the total genetic effects of the BLs in these locations were similar.

Total genetic effects of predicted superior line (+) of upper leaf angle were similar by using full model (G + GE ≙ 17.53°) and additive model (G ≙ 17.65°), because there were small impacts of dominance and epistasis effects on ULA (only two loci with dominance effects and three pairs of epistasis highly significantly identified). The predicted genetic effects of superior line (-) were smaller in full model (G ≙ -26.54 mm for LW and -167.46 mm for LL) as compared to additive model (G ≙ -19.99 mm for LW_A and -131.86 mm for LL_A). The genotypes for the identified loci corresponding to several predicted lines were presented in Supplementary Table S4 that might help to maize breeders.

## Conclusion

This study provided a demonstration of usefulness of full genetic model over additive model for analyzing complex plant traits and explored new insights about complex genetic architecture of the maize leaf traits. We predicted genetic potentials of the current BLs, SL, SH that could be useful for trait improvement.

## Author Contributions

JZ developed the analysis methods and analyzed the data. MM wrote the manuscript.

## Conflict of Interest Statement

The authors declare that the research was conducted in the absence of any commercial or financial relationships that could be construed as a potential conflict of interest.

## Footnotes

1 http://ensembl.gramene.org/Zea_mays/

2 http://www.uniprot.org/uniprot/C4J0G4
