# Supplementary material for: Dominance and Epistasis Interactions Revealed as Important Variants for Leaf Traits of Maize NAM Population
Source: Front Plant Sci. 2018 Jun 18;9:627. doi: 10.3389/fpls.2018.00627 (PMC6015889; doi:10.3389/fpls.2018.00627)
Supplement: Supplementary file 1 [file Table_1.DOC]

**Table S1 | Estimated genetic effects, standard errors, −log_10_*P_EW_*, and heritability of full model for leaf angle of maize.**

| QTS | Gene | Effect | Estimate | SE | −log_10_P*_EW_* | | $h^{2}$% | | Gene Descriptions | |
| --- | --- | --- | --- | --- | --- | --- | --- | --- | --- | --- |
| S1_19944329 | GRMZM2G006781 | *a* | 0.431 | 0.039 | 27.0 | | 0.74 | | Uncharacterized protein | |
| S1_35133062 | *GRMZM2G136513* | *a* | 0.841 | 0.039 | 99.7 | | 2.82 | | Unknown | |
|  |  | *ae_2_* | 0.389 | 0.075 | 6.7 | | 0.58 | |  | |
|  |  | *ae_3_* | -0.376 | 0.074 | 6.4 | |  | |
| S1_44956540 | AC212219.3_FG005 | *a* | 0.262 | 0.039 | 10.8 | | 0.27 | | Uncharacterized protein | |
| S1_258657782 | GRMZM2G108766 | *a* | 0.718 | 0.039 | 73.0 | | 2.06 | | Tubulin beta-3 chain | |
|  |  | *ae_1_* | 0.355 | 0.080 | 5.1 | | 0.50 | |  | |
| S1_273371612 | GRMZM2G180232 | *a* | 0.325 | 0.039 | 15.9 | | 0.42 | | Unknown | |
| S2_7180843 | GRMZM2G115716 | *a* | 0.259 | 0.039 | 10.4 | | 0.27 | | Unknown | |
| S2_62991412 | GRMZM2G014695 | *a* | 0.459 | 0.040 | 30.3 | | 0.84 | | Uncharacterized protein | |
| S3_162063504 | GRMZM2G180406 | *a* | -0.308 | 0.040 | 13.9 | | 0.38 | | Uncharacterized protein | |
| S3_163994316 | GRMZM5G801369 | *a* | -0.505 | 0.040 | 35.4 | | 1.01 | | Lysine ketoglutarate reductase trans-splicing related 1 | |
| S3_171437505 | GRMZM2G110153 | *ae_4_* | -0.653 | 0.080 | 15.6 | | 1.70 | | Putative MADS-domain transcription factor | |
| S3_177144018 | GRMZM2G396231 | *a* | -0.447 | 0.040 | 28.0 | | 0.80 | | Unknown | |
| S3_177492233 | GRMZM2G053801 | *a* | -0.673 | 0.040 | 63.8 | | 1.80 | | G10-like protein | |
| S3_216647852 | GRMZM2G464976 | *a* | 0.712 | 0.039 | 72.4 | | 2.02 | | Uncharacterized protein | |
| S4_185870953 | GRMZM5G840955 | *a* | 0.417 | 0.039 | 25.7 | | 0.69 | | Uncharacterized protein | |
| S5_23504867 | GRMZM5G816015 | *a* | -0.275 | 0.040 | 11.1 | | 0.30 | | Uncharacterized protein | |
| S5_24377120 | GRMZM2G135940 | *a* | 0.404 | 0.039 | 23.9 | | 0.65 | | Uncharacterized protein | |
| S5_29918641 | GRMZM2G066578 | *a* | 0.802 | 0.039 | 92.0 | | 2.56 | | Uncharacterized protein | |
| S5_32090529 | AC233949.1_FG004 | *a* | 0.297 | 0.039 | 13.5 | | 0.35 | | Cell division cycle protein 48 | |
| S5_35629887 | GRMZM2G065268 | *ae_1_* | -0.576 | 0.082 | 11.7 | | 0.94 | | Unknown | |
|  |  | *ae_3_* | 0.384 | 0.075 | 6.5 | |  | |
|  |  | *ae_4_* | 0.416 | 0.081 | 6.6 | |  | |
| S5_63801506 | *GRMZM5G834758* | *a* | 0.549 | 0.039 | 43.4 | | 1.20 | | DNA-binding protein MNB1B | |
| S5_65225359 | GRMZM2G055682 | *a* | 0.520 | 0.040 | 38.6 | | 1.08 | | CONTAINS InterPro DOMAIN/s: mRNA splicing factor | |
|  | (Cwf18) | *ae_4_* | 0.581 | 0.080 | 12.3 | | 1.35 | |  | |
| S5_84825303 | GRMZM2G382914 | *a* | 0.781 | 0.040 | 83.2 | | 2.43 | | Phosphoglycerate kinase | |
|  |  | *ae_1_* | 0.531 | 0.083 | 9.8 | | 0.98 | |  | |
|  |  | *ae_2_* | -0.457 | 0.076 | 8.6 | |  | |
| S5_98223550 | GRMZM2G134393 | *a* | -0.209 | 0.040 | 6.8 | | 0.17 | | Unknown | |
| S5_194091993 | GRMZM2G013821 | *a* | -0.598 | 0.040 | 50.6 | | 1.42 | | HMGc1 protein | |
| S5_201069419 | GRMZM2G050734 | *a* | 0.702 | 0.040 | 68.6 | | 1.96 | | Ubiquitin-protein ligase | |
| S6_34565567 | GRMZM2G180519 | *a* | -0.301 | 0.039 | 13.9 | | 0.36 | | Uncharacterized protein | |
| S6_60149057 | GRMZM5G888886 | *a* | -0.559 | 0.039 | 44.6 | | 1.24 | | Uncharacterized protein | |
|  |  | *ae_1_* | -0.444 | 0.080 | 7.5 | | 0.78 | |  | |
| S8_77253417 | GRMZM2G430027 | *d* | 0.924 | 0.181 | 6.5 | 1.70 | | Uncharacterized protein | |  |
| S8_78857312 | GRMZM2G073774 | *a* | 0.400 | 0.039 | 23.5 | 0.64 | | Dihydrolipoyl dehydrogenase | |  |
| S8_166675138 | GRMZM2G363408 | *a* | -0.210 | 0.039 | 7.0 | 0.18 | | Uncharacterized protein | |  |
| S9_28046935 | GRMZM2G041668 | *a* | -0.327 | 0.040 | 15.8 | 0.42 | | ANAC076; Putative NAC domain transcription factor superfamily protein; Secondary wall NAC transcription factor 7 | |  |
| S9_109173000 | GRMZM2G103647 | *a* | -0.556 | 0.040 | 43.9 | 1.23 | | Light-inducible protein CPRF-2; Putative bZIP transcription factor superfamily protein | |  |
| S9_142035936 | GRMZM2G317895 | *a* | -0.292 | 0.040 | 12.5 | 0.34 | | Uncharacterized protein | |  |
| S9_146872957 | GRMZM2G143450 | *a* | -0.535 | 0.039 | 41.4 | 1.14 | | Uncharacterized protein | |  |
| S10_59115236 | GRMZM2G053177 | *a* | -0.295 | 0.039 | 13.1 | 0.35 | | Uncharacterized protein | |  |
| S10_60155825 | GRMZM2G079777 | *a* | 0.515 | 0.039 | 38.3 | 1.06 | | Vacuolar ATP synthase subunit D 1 | |  |
| S10_144934798 | GRMZM2G527256 | *a* | 0.504 | 0.039 | 37.0 | 1.01 | | Uncharacterized protein | |  |
| S3_171437505× | GRMZM2G110153 × | *aa* | 0.509 | 0.040 | 36.2 | 2.07 | | Putative MADS-domain transcription factor | |  |
| S10_60155825 | GRMZM2G079777 |  |  |  |  |  | | Vacuolar ATP synthase subunit D 1 | |  |
| S5_63801506× | GRMZM5G834758× | *aa* | 0.399 | 0.040 | 22.3 | 1.27 | | DNA-binding protein MNB1B | |  |
| S10_59115236 | GRMZM2G053177 |  |  |  |  |  | | Uncharacterized protein | |  |
| S8_63557902× | GRMZM5G822895 × | *aa* | 0.722 | 0.040 | 72.2 | 4.15 | | Uncharacterized protein | |  |
| S8_77253417 | GRMZM2G430027 |  |  |  |  |  | | Uncharacterized protein | |  |

QTS: identified quantitative trait SNP; Gene: near or holder gene ID collected from grammene database; Effect: type of gene effects; −log_10_P*_EW_:* minus log experimental-wise P-value; $h^{2}$%: estimated heritability for the effects; Gene Description: description of the candidate genes collected from NCBI gene database.
