# Supplementary material for: Dominance and Epistasis Interactions Revealed as Important Variants for Leaf Traits of Maize NAM Population
Source: Front Plant Sci. 2018 Jun 18;9:627. doi: 10.3389/fpls.2018.00627 (PMC6015889; doi:10.3389/fpls.2018.00627)
Supplement: Supplementary file 2 [file Table_2.DOC]

**Table S2 | Estimated genetic effects, standard errors, −log_10_*P_EW_*, and heritability of full model for leaf width of maize.**

| QTS | Gene | Effect | Estimate | SE | −log_10_P*_EW_* | | $h^{2}$% | | Gene Descriptions | |
| --- | --- | --- | --- | --- | --- | --- | --- | --- | --- | --- |
| S1_15791359 | GRMZM2G119933 | *a* | -0.781 | 0.077 | 23.5 | | 0.26 | | transferases;tRNA (5-methylaminomethyl-2-thiouridylate)-methyltransferases | |
| S1_30042877 | GRMZM2G110131 | *a* | -0.748 | 0.078 | 21.2 | | 0.23 | | PnFL-2; Putative tify domain/CCT motif transcription factor family protein | |
| S1_33287311 | GRMZM2G159110 | *a* | -1.208 | 0.077 | 53.8 | | 0.61 | | Uncharacterized protein | |
|  |  | *d* | -1.655 | 0.316 | 6.8 | | 0.58 | |  | |
| S1_108623483 | GRMZM2G036567 | *a* | 0.564 | 0.078 | 12.3 | | 0.13 | | Uncharacterized protein | |
| S1_193828461 | GRMZM2G148744 | *d* | -1.815 | 0.369 | 6.1 | | 0.69 | | Uncharacterized protein | |
| S1_253901998 | GRMZM2G043191 | *d* | 1.445 | 0.282 | 6.5 | | 0.44 | | inositol polyphosphate 5-phosphatase 11 | |
| S1_262183895 | GRMZM2G101856 | *a* | 1.378 | 0.077 | 70.1 | | 0.80 | | Unknown | |
| S1_284007049 | GRMZM5G811069 | *a* | -0.892 | 0.079 | 28.9 | | 0.33 | | methyltransferases | |
| S2_8912586 | GRMZM5G876898 | *a* | 0.668 | 0.077 | 17.2 | | 0.19 | | Aminomethyltransferase | |
| S2_48133878 | GRMZM2G017187 | *ae_1_* | -0.770 | 0.153 | 6.3 | | 0.35 | | Auxin response factor 6 | |
|  |  | *ae_3_* | 1.032 | 0.160 | 10.0 | |  | |
| S2_86793193 | GRMZM2G052268 | *a* | -0.772 | 0.078 | 22.4 | | 0.25 | | F-box domain containing protein | |
| S2_105719757 | GRMZM2G067000 | *a* | -0.512 | 0.078 | 10.3 | | 0.11 | | Unknown | |
| S2_211163476 | GRMZM2G021567 | *a* | 0.795 | 0.077 | 24.3 | | 0.27 | | Pentatricopeptide repeat (PPR) superfamily protein | |
| S3_167364742 | GRMZM2G093119 | *a* | 0.964 | 0.077 | 34.7 | | 0.39 | | Uncharacterized protein | |
|  |  | *ae_2_* | 0.676 | 0.145 | 5.5 | | 0.19 | |  | |
| S3_174535977 | GRMZM2G129428 | *d* | -3.511 | 0.417 | 16.4 | | 2.59 | | Uncharacterized protein | |
|  |  | *ae_1_* | 0.857 | 0.152 | 7.8 | | 0.44 | |  | |
|  |  | *ae_2_* | -1.166 | 0.147 | 14.7 | |  | |
| S3_179228490 | GRMZM2G005112 | *a* | -0.522 | 0.078 | 10.7 | | 0.11 | | Unknown | |
| S5_4154084 | AC191048.2_FG006 | *a* | 0.891 | 0.078 | 29.7 | | 0.33 | | Prenylated Rab receptor 2 | |
| S5_14472560 | GRMZM2G006329 | *a* | 0.858 | 0.078 | 27.4 | | 0.31 | | PH domain containing protein | |
| S5_17891197 | GRMZM2G046676 | *a* | -0.565 | 0.077 | 12.6 | | 0.13 | | Charged multivesicular body protein 2a | |
| S5_23573289 | GRMZM2G074543 | *a* | -0.542 | 0.078 | 11.4 | | 0.12 | | Yabby9 protein | |
|  | (Yabby9) | *ae_4_* | 1.025 | 0.151 | 10.9 | | 0.44 | |  | |
| S5_24975010 | GRMZM2G374385 | *a* | -0.483 | 0.078 | 9.3 | | 0.10 | | Noc2p family | |
|  |  | *ae_4_* | 1.191 | 0.150 | 14.6 | | 0.60 | |  | |
| S5_27949015 | GRMZM2G159399 | *a* | 1.129 | 0.078 | 46.8 | | 0.54 | | Uncharacterized protein | |
|  |  | *ae_2_* | 0.774 | 0.147 | 6.8 | | 0.25 | |  | |
| S5_33980512 | GRMZM2G075150 | *a* | -1.289 | 0.077 | 61.2 | | 0.70 | | Uncharacterized protein | |
| S5_36520884 | GRMZM2G110976 | *a* | -0.914 | 0.077 | 32.0 | | 0.35 | | Unknown | |
|  |  | *ae_2_* | -0.654 | 0.147 | 5.1 | | 0.28 | |  | |
|  |  | *ae_4_* | 0.937 | 0.149 | 9.5 | |  | |
| S5_60282370 | GRMZM2G108775 | *ae_2_* | 0.971 | 0.146 | 10.5 | 0.40 | | FMN-linked oxidoreductases superfamily protein | |  |
| S5_61672558 | GRMZM2G300133 (DWARF1) | *a* | -0.984 | 0.077 | 36.3 | 0.41 | | Leucine-rich repeat receptor-like kinase protein THICK TASSEL DWARF1 | |  |
| S5_65208138 | GRMZM2G055619 | *a* | -0.919 | 0.078 | 31.3 | 0.35 | | Unknown | |  |
| S5_133333397 | GRMZM2G105494 | *a* | 0.748 | 0.077 | 21.4 | 0.23 | | Uncharacterized protein | |  |
| S5_146414091 | GRMZM2G064558 | *a* | -0.612 | 0.077 | 14.8 | 0.16 | | Unknown | |  |
|  |  | *d* | 1.761 | 0.385 | 5.3 | 0.65 | |  | |  |
| S5_201392548 | GRMZM2G140694 | *a* | 0.674 | 0.076 | 18.0 | 0.19 | | Uncharacterized protein | |  |
|  |  | *d* | -2.067 | 0.455 | 5.3 | 0.90 | |  | |  |
| S6_106344079 | GRMZM2G071638 | *a* | 1.409 | 0.077 | 73.8 | 0.83 | | Putative BCR family protein | |  |
| S6_143981358 | GRMZM2G424582 | *a* | 1.369 | 0.077 | 69.0 | 0.79 | | Uncharacterized protein | |  |
| S6_159891173 | GRMZM2G029546 | *a* | -0.385 | 0.077 | 6.2 | 0.06 | | Electron transporter/ heat shock protein binding protein | |  |
| S7_1952249 | GRMZM2G019200 | *a* | 0.677 | 0.077 | 17.7 | 0.19 | | calmodulin binding;purine nucleotide binding | |  |
| S8_44551961 | GRMZM2G012601 | *a* | 0.867 | 0.077 | 28.5 | 0.32 | | Putative ulp1 protease family protein | |  |
|  | (Ulp1) | *ae_2_* | -0.838 | 0.147 | 7.9 | 0.28 | |  | |  |
|  |  | *ae_4_* | 0.782 | 0.150 | 6.8 |  | |  |
| S8_63537498 | GRMZM5G822895 | *a* | -1.066 | 0.077 | 42.4 | 0.48 | | Uncharacterized protein | |  |
| S8_65750670 | GRMZM2G037345 | *a* | 0.800 | 0.077 | 24.3 | 0.27 | |  | |  |
| S8_88438999 | GRMZM2G057116 | *ae_2_* | 0.720 | 0.145 | 6.2 | 0.25 | | Putative WRKY DNA-binding domain superfamily protein | |  |
|  |  | *ae_4_* | -0.818 | 0.147 | 7.6 |  | |  |
| S8_121669693 | GRMZM2G060327 | *a* | 0.562 | 0.077 | 12.5 | 0.13 | | Uncharacterized protein | |  |
| S8_125301167 | GRMZM2G034551 | *a* | 1.578 | 0.076 | 93.5 | 1.05 | | ZIP metal ion transporter family | |  |
|  |  | *ae_1_* | 2.104 | 0.152 | 42.5 | 1.11 | |  | |  |
|  |  | *ae_3_* | -1.654 | 0.159 | 24.6 |  | |  |
|  |  | *ae_4_* | -0.882 | 0.150 | 8.4 |  | |  |
| S9_47763086 | GRMZM2G062618 | *a* | -0.644 | 0.077 | 16.1 | 0.17 | | Uncharacterized protein | |  |
| S9_109173783 | GRMZM2G103647 | *a* | -0.591 | 0.077 | 13.7 | 0.15 | | Light-inducible protein CPRF-2; Putative bZIP transcription factor superfamily protein | |  |
| S9_118717459 | GRMZM2G113873 | *a* | -0.838 | 0.078 | 26.5 | 0.30 | | Uncharacterized protein | |  |
| S9_146873044 | GRMZM2G143450 | *a* | -0.753 | 0.077 | 21.6 | 0.24 | | Uncharacterized protein | |  |
| S10_141070659 | GRMZM2G155949 | *a* | 1.177 | 0.076 | 52.5 | 0.58 | | Uncharacterized protein | |  |
| S1_262183895× | GRMZM2G101856× | *aa* | -1.124 | 0.078 | 46.4 | 1.06 | | Unknown | |  |
| S8_88438999 | GRMZM2G154881 | *da* | 1.773 | 0.371 | 5.8 | 1.32 | | 1,4-beta-D-glucanase; Endo-1,3;1,4-beta-D-glucanase; | |  |
| S1_284007049× | GRMZM2G454830× | *aa* | 0.404 | 0.081 | 6.3 | 0.14 | | Unknown | |  |
| S9_146873044 | GRMZM2G143450 | *dd* | 3.122 | 0.619 | 6.3 | 2.05 | | Uncharacterized protein | |  |
| S2_48133878× | GRMZM2G017187 × | *aa* | -0.462 | 0.080 | 8.2 | 0.18 | | Auxin response factor 6 | |  |
| S2_86793193 | GRMZM2G052268 | *da* | 1.956 | 0.364 | 7.1 | 1.61 | | F-box domain containing protein | |  |
| S2_79769999× | GRMZM2G102699 × | *aa* | -0.628 | 0.080 | 14.3 | 0.33 | | Uncharacterized protein | |  |
| S5_23573289 | GRMZM2G074543 (Yabby9) | *dd* | 5.078 | 0.728 | 11.5 | 5.42 | | Yabby9 protein | |  |
| S2_86793193× | GRMZM2G052268 × | *aa* | 0.777 | 0.080 | 21.3 | 0.51 | | F-box domain containing protein | |  |
| S5_23573289 | GRMZM2G074543 (Yabby9) | |  |  |  |  | | Yabby9 protein | |  |
| S3_174535977× | GRMZM2G129428 × | *aa* | 0.502 | 0.078 | 10.0 | 0.21 | | Uncharacterized protein | |  |
| S5_60282370 | GRMZM2G108775 | *da* | 2.400 | 0.487 | 6.1 | 2.42 | | FMN-linked oxidoreductases superfamily protein | |  |
|  |  | *dd* | 7.562 | 0.807 | 20.1 | 12.01 | |  | |  |
| S3_174535977× | GRMZM2G129428 × | *aa* | 1.059 | 0.078 | 41.0 | 0.94 | | Uncharacterized protein | |  |
| S6_159891173 | GRMZM2G029546 | *ad* | 1.765 | 0.372 | 5.7 | 1.31 | | Electron transporter/ heat shock protein binding protein | |  |
|  |  | *da* | 2.792 | 0.493 | 7.8 | 3.28 | |  | |  |
| S5_32095057× | AC233949.1_FG004× | *aa* | 0.386 | 0.079 | 6.0 | 0.12 | | Cell division cycle protein 48 | |  |
| S5_65208138 | GRMZM2G057000 | *ad* | 1.543 | 0.326 | 5.7 | 1.00 | | Brassinosteroid biosynthesis-like protein | |  |
| S5_201392548× | GRMZM2G140694× | *da* | 3.790 | 0.549 | 11.3 | 6.04 | | Uncharacterized protein | |  |
| S6_143981358 | GRMZM2G061143 |  |  |  |  |  | | Uncharacterized protein | |  |

QTS: identified quantitative trait SNP; Gene: near or holder gene ID collected from grammene database; Effect: type of gene effects; −log_10_P*_EW_:* minus log experimental-wise P-value; $h^{2}$%: estimated heritability for the effects; Gene Description: description of the candidate genes collected from NCBI gene database.
