# Supplementary material for: Dominance and Epistasis Interactions Revealed as Important Variants for Leaf Traits of Maize NAM Population
Source: Front Plant Sci. 2018 Jun 18;9:627. doi: 10.3389/fpls.2018.00627 (PMC6015889; doi:10.3389/fpls.2018.00627)
Supplement: Supplementary file 3 [file Table_3.DOC]

**Table S3 | Estimated genetic effects, standard errors, −log_10_*P_EW_*, and heritability** of full model for leaf length of maize.

| QTS | Gene ID | Effect | Estimate | SE | −log_10_P*_EW_* | | $h^{2}$% | | Gene Description | |  |
| --- | --- | --- | --- | --- | --- | --- | --- | --- | --- | --- | --- |
| S1_22592882 | AC177837.3_FG002 | *a* | -3.990 | 0.546 | 12.5 | 0.13 | | | Unknown | |  |
|  |  | *ae_3_* | 6.322 | 1.130 | 7.6 | 0.32 | | |  | |  |
| S1_30042877 | GRMZM2G110131 | *a* | 4.783 | 0.551 | 17.4 | 0.18 | | | PnFL-2; Putative tify domain/CCT motif transcription factor family protein | |  |
| S1_38610159 | GRMZM2G128444 | *a* | -8.513 | 0.542 | 54.5 | 0.57 | | | Cysteine proteinases superfamily protein | |  |
| S1_44373499 | GRMZM2G107867 | *a* | 2.952 | 0.551 | 7.1 | 0.07 | | | SNF1-related protein kinase | |  |
| S1_236903080 | GRMZM5G810570 | *a* | -3.673 | 0.549 | 10.6 | 0.11 | | | Unknown | |  |
| S1_251103220 | GRMZM2G127546 (E1) | *a* | -3.841 | 0.543 | 11.8 | 0.12 | | | Pyruvate dehydrogenase E1 component subunit beta | |  |
| S2_3410957 | GRMZM2G348909 | *a* | 6.192 | 0.559 | 27.7 | 0.30 | | | Uncharacterized protein | |  |
|  |  | *ae_3_* | -9.065 | 1.168 | 14.0 | 0.46 | | |  | |  |
|  |  | *ae_4_* | 5.785 | 1.082 | 7.0 |  | |  |
| S2_13990360 | GRMZM2G100732 | *a* | 8.066 | 0.549 | 47.9 | 0.51 | | | Unknown | |  |
| S2_79769999 | GRMZM2G102699 | *a* | -2.976 | 0.551 | 7.2 | 0.07 | | | Uncharacterized protein | |  |
| S2_175085392 | GRMZM2G108698 | *a* | 6.049 | 0.553 | 27.0 | 0.29 | | | Unknown | |  |
| S2_211163476 | GRMZM2G021567 | *a* | 3.589 | 0.546 | 10.3 | 0.10 | | | Pentatricopeptide repeat (PPR) superfamily protein | |  |
| S3_35995225 | GRMZM2G120115 | *a* | 6.476 | 0.546 | 31.7 | 0.33 | | | Uncharacterized protein | |  |
| S3_114352354 | GRMZM2G105991 | *a* | -6.944 | 0.545 | 36.3 | 0.38 | | | Uncharacterized protein | |  |
| S3_167364742 | GRMZM2G093119 | *a* | 9.177 | 0.550 | 61.4 | 0.67 | | | Uncharacterized protein | |  |
|  |  | *d* | 11.370 | 2.226 | 6.5 | 0.51 | | |  | |  |
|  |  | *ae_3_* | -5.318 | 1.128 | 5.6 | 0.22 | | |  | |  |
| S3_180498932 | GRMZM2G064853 | *d* | 22.330 | 2.528 | 18.0 | 1.97 | | | Uncharacterized protein | |  |
| S3_201013951 | GRMZM2G166759 | *a* | -7.948 | 0.548 | 46.7 | 0.50 | | | Unknown | |  |
| S3_218013991 | GRMZM2G057733 | *a* | 5.756 | 0.551 | 24.8 | 0.26 | | | Transmembrane amino acid transporter family protein | |  |
| S4_5406854 | GRMZM2G154414 | *a* | -2.987 | 0.550 | 7.2 | 0.07 | | | Uncharacterized protein | |  |
| S4_153318619 | GRMZM2G463471 | *a* | -7.529 | 0.546 | 42.3 | 0.45 | | | Actin-depolymerizing factor | |  |
| S5_3772033 | GRMZM2G054350 | *a* | 6.704 | 0.548 | 33.5 | 0.36 | | | Unknown | |  |
| S5_24218177 | AC196432.3_FG007 | *a* | -9.986 | 0.548 | 72.8 | 0.79 | | | Unknown | |  |
| S5_29917954 | GRMZM2G066578 | *a* | 5.627 | 0.549 | 23.8 | 0.25 | | | Uncharacterized protein | |  |
| S5_56214402 | GRMZM2G043348 | *a* | -2.507 | 0.564 | 5.1 | 0.05 | | | Uncharacterized protein | |  |
| S5_58606840 | *GRMZM5G854533* | *a* | 4.159 | 0.557 | 13.1 | 0.14 | | | chloroplast thylakoid lumen protein | |  |
|  |  | *d* | 12.308 | 1.873 | 10.3 | 0.60 | | |  | |  |
| S5_74622667 | GRMZM2G168747 | *ae3* | -6.858 | 1.171 | 8.3 | 0.34 | | | Unknown | |  |
|  |  | *ae_4_* | 6.218 | 1.087 | 8.0 |  | |  |
| S5_82557231 | GRMZM2G005562 | *a* | 5.001 | 0.548 | 19.1 | 0.20 | | | Pectin lyase-like superfamily protein | |  |
| S5_162835530 | GRMZM5G870269 | *d* | -10.223 | 1.795 | 7.9 | 0.41 | | | Unknown | |  |
| S5_175592302 | GRMZM2G049675 | *a* | 3.416 | 0.559 | 9.0 | 0.09 | | | Gibberellin receptor GID1L2 | |  |
|  | (GID1L2) | *ae_4_* | 5.312 | 1.053 | 6.3 | 0.22 | | |  | |  |
| S6_58441439 | GRMZM2G103230 | *a* | 10.578 | 0.542 | 83.4 | | | 0.89 | | Uncharacterized protein | |
|  |  | *ae_3_* | -6.803 | 1.126 | 8.8 | | | 0.37 | |  | |
| S6_162119944 | GRMZM2G093346 | *a* | -11.479 | 0.556 | 93.1 | | | 1.04 | | Cytosolic Ascorbate Peroxidase 1 | |
| S7_131033571 | GRMZM2G459503 | *a* | 5.383 | 0.545 | 22.3 | | | 0.23 | | Uncharacterized protein | |
| S7_156118077 | GRMZM2G116603 | *a* | -2.417 | 0.545 | 5.0 | | | 0.05 | | F-box domain containing protein | |
|  |  | *ae_4_* | 5.936 | 1.038 | 8.0 | | | 0.28 | |  | |
| S8_23427828 | GRMZM2G472625 | *a* | 2.518 | 0.552 | 5.3 | | | 0.05 | | Putative glycogen synthase kinase family protein | |
|  |  | *ae_4_* | 5.228 | 1.044 | 6.3 | | | 0.22 | |  | |
| S8_79142489 | GRMZM2G040467 | *a* | 11.424 | 0.540 | 97.4 | | | 1.03 | | Uncharacterized protein | |
| S8_152131943 | GRMZM2G060886 | *a* | 9.448 | 0.551 | 64.7 | | | 0.71 | | Uncharacterized protein | |
|  |  | *d* | -10.107 | 2.153 | 5.6 | | | 0.40 | |  | |
|  |  | *ae_1_* | 5.689 | 1.084 | 6.8 | | | 0.41 | |  | |
|  |  | *ae_3_* | 5.770 | 1.157 | 6.2 | | |  | |
|  |  | *ae_4_* | -9.558 | 1.070 | 18.4 | | |  | |
| S9_2825568 | GRMZM5G863029 | *a* | -4.217 | 0.554 | 13.6 | | | 0.14 | | Uncharacterized protein | |
| S9_44334377 | GRMZM2G110993 | *a* | 3.469 | 0.550 | 9.5 | | | 0.10 | | Putative endo-1,4-beta-mannosidase family protein | |
|  |  | *d* | 12.080 | 2.219 | 7.3 | | | 0.58 | |  | |
| S9_100789144 | GRMZM2G092586 | *ae_4_* | -5.643 | 1.042 | 7.2 | | | 0.25 | | Uncharacterized protein | |
| S9_109173000 | GRMZM2G103647 | *a* | 5.955 | 0.552 | 26.3 | | | 0.28 | | Light-inducible protein CPRF-2; Putative bZIP transcription factor superfamily protein | |
| S9_142470352 | GRMZM2G031441 | *a* | 6.891 | 0.552 | 34.9 | | | 0.38 | | Putative homeodomain-like transcription factor superfamily protein | |
| S10_135678209 | GRMZM2G086474 | *a* | -6.504 | 0.557 | 30.6 | | | 0.33 | | Putative HLH DNA-binding domain superfamily protein | |
| S1_30042877× | GRMZM2G110131× | *aa* | -3.257 | 0.564 | 8.1 | | | 0.17 | | PnFL-2; Putative tify domain/CCT motif transcription factor family protein | |
| S1_187636354 | GRMZM2G132763 |  |  |  |  | | |  | | Unknown | |
| S1_30042877× | GRMZM2G110131× | *aa* | -3.528 | 0.562 | 9.5 | | | 0.20 | | PnFL-2; Putative tify domain/CCT motif transcription factor family protein | |
| S3_180498932 | GRMZM2G064853 | *dd* | -38.732 | 5.685 | 11.0 | | | 5.93 | | Uncharacterized protein | |
| S1_38610159× | GRMZM2G128444 × | *aa* | -5.912 | 0.555 | 25.7 | | | 0.55 | | Cysteine proteinases superfamily protein | |
| S9_100789144 | GRMZM2G092586 |  |  |  |  | | |  | | Uncharacterized protein | |
| S1_75631383× | GRMZM5G852170 × | *aa* | -4.642 | 0.565 | 15.7 | | | 0.34 | | Unknown | |
| S9_98495768 | GRMZM2G177659 |  |  |  |  | | |  | | Amino acid transporter-like protein | |
| S1_75631383× | GRMZM5G852170 × | *aa* | 8.832 | 0.562 | 54.7 | | | 1.23 | | Unknown | |
| S10_116425230 | GRMZM2G022382 |  |  |  |  | | |  | | Uncharacterized protein | |
| S1_251103220× | GRMZM2G127546 × | *aa* | 5.362 | 0.555 | 21.3 | | | 0.45 | | Pyruvate dehydrogenase E1 component subunit beta | |
| S5_24218177 | AC196432.3_FG007 | *dd* | 45.052 | 5.282 | 16.8 | | | 8.03 | | Unknown | |
|  |  | *aae_4_* | -5.429 | 1.040 | 6.7 | | | 0.47 | |  | |
| S1_251103220× | GRMZM2G127546 (E1) × | *aa* | 8.368 | 0.563 | 49.0 | | | 1.11 | | Pyruvate dehydrogenase E1 component subunit beta | |
| S6_162119944 | GRMZM2G093346 | *da* | -21.528 | 3.503 | 9.1 | | | 3.67 | | Uncharacterized protein | |
| S2_175085392× | GRMZM2G108698 × | *aa* | -4.574 | 0.566 | 15.1 | | | 0.33 | | Unknown | |
| S3_167364742 | GRMZM2G093119 | *dd* | -30.583 | 4.466 | 11.1 | | | 3.70 | | Uncharacterized protein | |
| S3_114352354× | GRMZM2G105991× | *aa* | -2.565 | 0.556 | 5.4 | | | 0.10 | | Uncharacterized protein | |
| S8_27221054 | GRMZM2G010596 | *dd* | -35.694 | 4.943 | 12.3 | | | 5.04 | | 3-methyl-2-oxobutanoate hydroxymethyltransferase | |
| S3_167364742× | GRMZM2G093119× | *aa* | 5.025 | 0.560 | 18.5 | | | 0.40 | | Uncharacterized protein | |
| S4_153318619 | GRMZM2G463471 |  |  |  |  | | |  | | Actin-depolymerizing factor | |
| S5_3772033× | GRMZM2G054350× | *aa* | 5.706 | 0.558 | 23.8 | | | 0.52 | | Unknown | |
| S7_156118077 | GRMZM2G116603 |  |  |  |  | | |  | | F-box domain containing protein | |
| S6_58441439× | GRMZM2G103230× | *aa* | -10.700 | 0.557 | 80.8 | | | 1.81 | | Uncharacterized protein | |
| S8_152131943 | GRMZM2G060886 | *dd* | 25.941 | 5.580 | 5.5 | | | 2.66 | | Uncharacterized protein | |
| S7_131033571× | GRMZM2G459503 × | *aa* | 2.545 | 0.560 | 5.3 | | | 0.10 | | Uncharacterized protein | |
| S9_142470352 | GRMZM2G031441 | *ad* | 17.748 | 2.313 | 13.8 | | | 2.49 | | Putative homeodomain-like transcription factor superfamily protein | |

QTS: identified quantitative trait SNP; Gene: near or holder gene ID collected from grammene database; Effect: type of gene effects; −log_10_P*_EW_:* minus log experimental-wise *P*-value; $h^{2}$%: estimated heritability for the effects; Gene Description: description of the candidate genes collected from NCBI gene database.
