# Supplementary material for: Dominance and Epistasis Interactions Revealed as Important Variants for Leaf Traits of Maize NAM Population
Source: Front Plant Sci. 2018 Jun 18;9:627. doi: 10.3389/fpls.2018.00627 (PMC6015889; doi:10.3389/fpls.2018.00627)
Supplement: Supplementary file 4 [file Table_4.DOC]

**Table S4 | Genotypic alleles of superior lines and hybrids for three leaf traits of maize.**

| Leaf Angle_F | Z014E0005 | GSL(+) | SL(+)1 | SL(+)2 | SL(+)3 | SL(+)4 | GSH(+) | SH(+)1 | SH(+)2 | SH(+)3 | SH(+)4 |
| --- | --- | --- | --- | --- | --- | --- | --- | --- | --- | --- | --- |
| S1_44956540 | *QQ* | *QQ* | *QQ* | *QQ* | *QQ* | *qq* | *Qq* | *Qq* | *Qq* | *Qq* | *Qq* |
| S1_273371612 | *qq* | *QQ* | *QQ* | *QQ* | *QQ* | *QQ* | *QQ* | *QQ* | *QQ* | *QQ* | *QQ* |
| S3_116026935 | *QQ* | *QQ* | *QQ* | *QQ* | *QQ* | *QQ* | *Qq* | *Qq* | *Qq* | *Qq* | *Qq* |
| S3_216647852 | *QQ* | *QQ* | *QQ* | *QQ* | *QQ* | *QQ* | *Qq* | *Qq* | *Qq* | *Qq* | *QQ* |
| S5_24377120 | *qq* | *QQ* | *QQ* | *QQ* | *QQ* | *QQ* | *Qq* | *Qq* | *Qq* | *Qq* | *Qq* |
| S5_35629887 | *qq* | *QQ* | *qq* | *qq* | *QQ* | *QQ* | *QQ* | *qq* | *qq* | *QQ* | *QQ* |
| S5_43741243 | *qq* | *QQ* | *QQ* | *QQ* | *QQ* | *QQ* | *QQ* | *QQ* | *Qq* | *QQ* | *QQ* |
| S5_60999978 | *qq* | *QQ* | *QQ* | *QQ* | *QQ* | *QQ* | *Qq* | *Qq* | *Qq* | *Qq* | *Qq* |
| S5_63801506 | *QQ* | *QQ* | *QQ* | *QQ* | *QQ* | *QQ* | *Qq* | *Qq* | *Qq* | *Qq* | *Qq* |
| S8_37237383 | *qq* | *QQ* | *QQ* | *QQ* | *QQ* | *QQ* | *QQ* | *QQ* | *QQ* | *QQ* | *QQ* |
| S8_63557902 | *QQ* | *QQ* | *QQ* | *QQ* | *QQ* | *QQ* | *Qq* | *Qq* | *Qq* | *Qq* | *QQ* |
| S8_77253417 | *QQ* | *QQ* | *QQ* | *QQ* | *QQ* | *QQ* | *Qq* | *Qq* | *Qq* | *Qq* | *QQ* |
| S8_166675138 | *qq* | *QQ* | *QQ* | *QQ* | *QQ* | *QQ* | *QQ* | *QQ* | *QQ* | *QQ* | *QQ* |
| S10_59115236 | *QQ* | *QQ* | *QQ* | *QQ* | *QQ* | *QQ* | *Qq* | *Qq* | *Qq* | *Qq* | *Qq* |
| S10_144934798 | *QQ* | *QQ* | *QQ* | *QQ* | *QQ* | *QQ* | *Qq* | *Qq* | *Qq* | *Qq* | *Qq* |
| Leaf Width_F | Z024E0055 | GSL(+) | SL(+)1 | SL(+)2 | SL(+)3 | SL(+)4 | GSH(+) | SH(+)1 | SH(+)2 | SH(+)3 | SH(+)4 |
| S1_33287311 | *QQ* | *QQ* | *QQ* | *QQ* | *QQ* | *QQ* | *Qq* | *Qq* | *Qq* | *Qq* | *Qq* |
| S1_108623483 | *qq* | *QQ* | *QQ* | *QQ* | *QQ* | *QQ* | *QQ* | *QQ* | *QQ* | *QQ* | *QQ* |
| S1_193828461 | *QQ* | *QQ* | *QQ* | *QQ* | *QQ* | *QQ* | *Qq* | *Qq* | *Qq* | *Qq* | *Qq* |
| S1_241404293 | *Qq* | *QQ* | *QQ* | *QQ* | *QQ* | *QQ* | *QQ* | *QQ* | *QQ* | *QQ* | *QQ* |
| S1_253901998 | *Qq* | *QQ* | *QQ* | *QQ* | *QQ* | *QQ* | *QQ* | *QQ* | *QQ* | *QQ* | *QQ* |
| S1_262183895 | *qq* | *qq* | *qq* | *qq* | *qq* | *qq* | *Qq* | *Qq* | *Qq* | *Qq* | *Qq* |
| S2_8912586 | *qq* | *QQ* | *QQ* | *QQ* | *QQ* | *QQ* | *QQ* | *QQ* | *QQ* | *QQ* | *QQ* |
| S2_48133878 | *Qq* | *QQ* | *QQ* | *QQ* | *qq* | *QQ* | *Qq* | *Qq* | *Qq* | *Qq* | *QQ* |
| S2_79769999 | *QQ* | *QQ* | *QQ* | *QQ* | *QQ* | *QQ* | *Qq* | *Qq* | *Qq* | *Qq* | *Qq* |
| S2_86793193 | *qq* | *qq* | *qq* | *qq* | *qq* | *QQ* | *Qq* | *Qq* | *Qq* | *Qq* | *QQ* |
| S3_174535977 | *qq* | *qq* | *qq* | *QQ* | *qq* | *qq* | *Qq* | *Qq* | *Qq* | *Qq* | *Qq* |
| S4_153318619 | *qq* | *QQ* | *QQ* | *QQ* | *QQ* | *QQ* | *QQ* | *QQ* | *QQ* | *QQ* | *QQ* |
| S5_23573289 | *QQ* | *QQ* | *QQ* | *QQ* | *QQ* | *qq* | *QQ* | *QQ* | *QQ* | *QQ* | *qq* |
| S5_24975010 | *qq* | *QQ* | *QQ* | *QQ* | *QQ* | *qq* | *QQ* | *QQ* | *QQ* | *QQ* | *qq* |
| S5_32095057 | *qq* | *QQ* | *QQ* | *QQ* | *QQ* | *QQ* | *QQ* | *QQ* | *QQ* | *QQ* | *QQ* |
| S5_36520884 | *qq* | *QQ* | *QQ* | *QQ* | *QQ* | *qq* | *QQ* | *QQ* | *QQ* | *QQ* | *qq* |
| S5_60282370 | *qq* | *QQ* | *QQ* | *qq* | *QQ* | *QQ* | *QQ* | *QQ* | *qq* | *QQ* | *QQ* |
| S5_61672558 | *Qq* | *QQ* | *QQ* | *QQ* | *QQ* | *QQ* | *QQ* | *QQ* | *QQ* | *QQ* | *QQ* |
| S5_65208138 | *Qq* | *QQ* | *QQ* | *QQ* | *QQ* | *QQ* | *QQ* | *QQ* | *QQ* | *QQ* | *QQ* |
| S5_133333397 | *qq* | *qq* | *qq* | *qq* | *qq* | *qq* | *Qq* | *Qq* | *Qq* | *Qq* | *Qq* |
| S5_201392548 | *qq* | *QQ* | *QQ* | *QQ* | *QQ* | *QQ* | *Qq* | *Qq* | *Qq* | *Qq* | *Qq* |
| S6_106344079 | *QQ* | *qq* | *qq* | *qq* | *qq* | *qq* | *qq* | *qq* | *qq* | *qq* | *qq* |
| S6_159891173 | *QQ* | *QQ* | *QQ* | *qq* | *QQ* | *QQ* | *Qq* | *Qq* | *Qq* | *Qq* | *Qq* |
| S7_1952249 | *qq* | *QQ* | *QQ* | *QQ* | *QQ* | *QQ* | *QQ* | *QQ* | *QQ* | *QQ* | *QQ* |
| S8_121669693 | *qq* | *QQ* | *QQ* | *QQ* | *QQ* | *QQ* | *QQ* | *QQ* | *QQ* | *QQ* | *QQ* |
| S8_125301167 | *qq* | *qq* | *qq* | *qq* | *QQ* | *qq* | *qq* | *qq* | *qq* | *QQ* | *qq* |
| S9_146873044 | *QQ* | *QQ* | *QQ* | *QQ* | *QQ* | *QQ* | *Qq* | *Qq* | *Qq* | *Qq* | *Qq* |
| Length_F | Z024E0103 | GSL(–) | SL(–)1 | SL(–)2 | SL(–)3 | SL(–)4 | GSH(–) | SH(–)1 | SH(–)2 | SH(–)3 | SH(–)4 |
| S1_30042877 | *QQ* | *QQ* | *QQ* | *QQ* | *QQ* | *QQ* | *Qq* | *Qq* | *Qq* | *Qq* | *Qq* |
| S1_38610159 | *QQ* | *QQ* | *QQ* | *QQ* | *QQ* | *QQ* | *Qq* | *Qq* | *Qq* | *Qq* | *QQ* |
| S1_75631383 | *QQ* | *qq* | *qq* | *qq* | *qq* | *qq* | *qq* | *qq* | *qq* | *qq* | *qq* |
| S1_187636354 | *QQ* | *QQ* | *QQ* | *QQ* | *QQ* | *QQ* | *Qq* | *Qq* | *Qq* | *Qq* | *Qq* |
| S1_251103220 | *qq* | *qq* | *qq* | *qq* | *qq* | *qq* | *Qq* | *Qq* | *Qq* | *Qq* | *Qq* |
| S2_3410957 | *qq* | *qq* | *qq* | *qq* | *QQ* | *qq* | *qq* | *qq* | *qq* | *QQ* | *qq* |
| S2_13990360 | *QQ* | *qq* | *qq* | *qq* | *qq* | *qq* | *qq* | *qq* | *qq* | *qq* | *qq* |
| S2_79769999 | *QQ* | *QQ* | *QQ* | *QQ* | *QQ* | *QQ* | *Qq* | *Qq* | *Qq* | *Qq* | *Qq* |
| S2_211163476 | *qq* | *QQ* | *QQ* | *QQ* | *QQ* | *QQ* | *QQ* | *QQ* | *QQ* | *QQ* | *QQ* |
| S3_180498932 | *QQ* | *QQ* | *QQ* | *QQ* | *QQ* | *QQ* | *Qq* | *Qq* | *Qq* | *Qq* | *Qq* |
| S4_153318619 | *qq* | *QQ* | *QQ* | *QQ* | *QQ* | *QQ* | *Qq* | *Qq* | *Qq* | *Qq* | *Qq* |
| S5_56214402 | *qq* | *QQ* | *QQ* | *QQ* | *QQ* | *QQ* | *QQ* | *QQ* | *QQ* | *QQ* | *QQ* |
| S5_58606840 | *qq* | *QQ* | *QQ* | *QQ* | *QQ* | *QQ* | *QQ* | *QQ* | *QQ* | *QQ* | *QQ* |
| S5_74622667 | *Qq* | *QQ* | *QQ* | *QQ* | *QQ* | *qq* | *QQ* | *QQ* | *QQ* | *QQ* | *qq* |
| S5_82557231 | *qq* | *QQ* | *QQ* | *QQ* | *QQ* | *QQ* | *QQ* | *QQ* | *QQ* | *QQ* | *QQ* |
| S5_162835530 | *Qq* | *QQ* | *QQ* | *QQ* | *QQ* | *QQ* | *Qq* | *Qq* | *Qq* | *Qq* | *Qq* |
| S5_175592302 | *qq* | *QQ* | *QQ* | *QQ* | *QQ* | *qq* | *QQ* | *QQ* | *QQ* | *QQ* | *qq* |
| S5_212157493 | *qq* | *QQ* | *QQ* | *QQ* | *QQ* | *QQ* | *QQ* | *QQ* | *QQ* | *QQ* | *QQ* |
| S6_58441439 | *qq* | *QQ* | *qq* | *QQ* | *qq* | *QQ* | *QQ* | *qq* | *QQ* | *qq* | *QQ* |
| S7_131033571 | *QQ* | *qq* | *qq* | *qq* | *qq* | *qq* | *qq* | *qq* | *qq* | *qq* | *qq* |
| S7_156118077 | *QQ* | *QQ* | *QQ* | *QQ* | *QQ* | *qq* | *QQ* | *QQ* | *QQ* | *QQ* | *qq* |
| S8_23427828 | *qq* | *QQ* | *QQ* | *QQ* | *QQ* | *qq* | *QQ* | *QQ* | *QQ* | *QQ* | *qq* |
| S8_152131943 | *qq* | *QQ* | *qq* | *QQ* | *qq* | *QQ* | *Qq* | *qq* | *Qq* | *qq* | *QQ* |
| S9_2825568 | *QQ* | *QQ* | *QQ* | *QQ* | *QQ* | *QQ* | *Qq* | *Qq* | *Qq* | *Qq* | *Qq* |
| S9_44334377 | *qq* | *QQ* | *QQ* | *QQ* | *QQ* | *QQ* | *QQ* | *QQ* | *QQ* | *QQ* | *QQ* |
| S9_98495768 | *qq* | *QQ* | *QQ* | *QQ* | *QQ* | *QQ* | *Qq* | *Qq* | *Qq* | *Qq* | *Qq* |
| S9_100789144 | *qq* | *QQ* | *QQ* | *QQ* | *QQ* | *QQ* | *Qq* | *Qq* | *Qq* | *Qq* | *QQ* |
| S9_142470352 | *QQ* | *qq* | *qq* | *qq* | *qq* | *qq* | *Qq* | *Qq* | *Qq* | *Qq* | *Qq* |
| S10_4879626 | *qq* | *QQ* | *QQ* | *QQ* | *QQ* | *QQ* | *QQ* | *QQ* | *QQ* | *QQ* | *QQ* |
| S10_116425230 | *qq* | *QQ* | *QQ* | *QQ* | *QQ* | *QQ* | *QQ* | *QQ* | *QQ* | *QQ* | *QQ* |
