# Supplementary material for: Dominance and Epistasis Interactions Revealed as Important Variants for Leaf Traits of Maize NAM Population
Source: Front Plant Sci. 2018 Jun 18;9:627. doi: 10.3389/fpls.2018.00627 (PMC6015889; doi:10.3389/fpls.2018.00627)
Supplement: Supplementary file 5 [file Table_5.DOC]

**Table S5 | Prediction of total genetic effects of leaf traits in additive model.**

| Entry | *G* | *G+GE1* | *G+GE2* | *G+GE3* | *G+GE4* |
| --- | --- | --- | --- | --- | --- |
| ULA_A (μ $\hat{=}$ 66.2209) |  |  |  |  |  |
| *QQ* | 0.21 | 0.67 | 0.77 | -0.64 | 0.04 |
| *qq* | -0.21 | -0.67 | -0.77 | 0.64 | -0.04 |
| Superior Line (+) | 13.23 | 15.50 | 13.47 | 11.53 | 14.20 |
| LW_A (μ $\hat{=}$90.9542) |  |  |  |  |  |
| *QQ* | 2.37 | 1.11 | 1.70 | 0.65 | 6.10 |
| *qq* | -2.37 | -1.11 | -1.70 | -0.65 | -6.10 |
| Superior Line (−) | -19.99 | -25.98 | -22.58 | -25.56 | -25.75 |
| LL_A (μ $\hat{=}$738.364) |  |  |  |  |  |
| *QQ* | 63.41 | 79.21 | 53.94 | 18.39 | 103.17 |
| *qq* | -63.41 | -79.21 | -53.94 | -18.39 | -103.17 |
| Superior Line (−) | -131.86 | -166.31 | -130.21 | -160.51 | -184.98 |
