# Supplementary material for: Dominance and Epistasis Interactions Revealed as Important Variants for Leaf Traits of Maize NAM Population
Source: Front Plant Sci. 2018 Jun 18;9:627. doi: 10.3389/fpls.2018.00627 (PMC6015889; doi:10.3389/fpls.2018.00627)
Supplement: Supplementary file 6 [file Table_6.DOC]

**Table S6 | Single simulation results of full model approach for the simulated trait controlling by only additive effects of loci**

| SNPID | Effect | Parameter | Estimate | SE | *P*_EW_-Value |
| --- | --- | --- | --- | --- | --- |
| S1_15791360 | *a* | -0.8307 | -0.734 | 0.0768 | 1.36E-21 |
| S1_33287311 | *a* | -1.1357 | -1.1638 | 0.0776 | 1.29E-50 |
| S1_44373499 | *a* | 0.8617 | 0.8124 | 0.0777 | 1.68E-25 |
| S1_193828461 | *a* | -0.3269 | -0.2575 | 0.077 | 8.21E-04 |
| S1_284007049 | *a* | -0.8435 | -0.8173 | 0.0789 | 4.32E-25 |
| S2_8912586 | *a* | 0.5814 | 0.5553 | 0.0775 | 8.19E-13 |
| S2_211163476 | *a* | 0.7557 | 0.8518 | 0.077 | 2.41E-28 |
| S3_167364742 | *a* | 1.1105 | 1.1753 | 0.0776 | 1.40E-51 |
| S3_179228490 | *a* | -0.6053 | -0.5209 | 0.0779 | 2.37E-11 |
| S4_5370399 | *a* | -0.4944 | 0.2389 | 0.078 | 2.21E-03 |
|  | *d* |  | -0.6719 | 0.2899 | 2.05E-02 |
| S5_14472560 | *a* | 0.8728 | 0.8428 | 0.0781 | 4.52E-27 |
| S5_23573289 | *a* | -0.8533 | -0.5501 | 0.0782 | 2.08E-12 |
| S5_32095057 | *a* |  | 0.4767 | 0.0769 | 5.86E-10 |
| S5_33980512 | *a* | -1.3132 | -1.4818 | 0.0775 | 8.88E-81 |
| S5_51435988 | *a* |  | -0.2673 | 0.0769 | 5.08E-04 |
| S5_58056878 | *a* | 0.703 | 0.6197 | 0.0783 | 2.56E-15 |
| S5_61672558 | *a* | -0.9859 | -0.9808 | 0.0773 | 1.05E-36 |
| S5_65208138 | *a* | -0.632 | -0.4684 | 0.0781 | 2.00E-09 |
|  | *de_1_* |  | -1.0733 | 0.5074 | 3.44E-02 |
|  | *de_3_* |  | 1.16 | 0.5543 | 3.64E-02 |
| S5_146414091 | *a* | -0.5577 | -0.7621 | 0.0768 | 3.85E-23 |
| S5_200435117 | *a* | 0.671 | 0.3566 | 0.077 | 3.60E-06 |
| S6_106344079 | *a* | 1.4285 | 1.2004 | 0.077 | 1.71E-54 |
| S6_143981358 | *a* | 1.4771 | 1.5683 | 0.0774 | 2.29E-90 |
| S7_1952249 | *a* | 0.6116 | 0.5768 | 0.0773 | 8.98E-14 |
|  | *d* |  | 1.0295 | 0.3355 | 2.15E-03 |
| S8_44399372 | *a* | 1.1075 | 0.9557 | 0.0765 | 1.22E-35 |
|  | *d* |  | -0.8675 | 0.4266 | 4.20E-02 |
| S8_63537498 | *a* | -0.7555 | -0.6905 | 0.0774 | 4.93E-19 |
|  | *d* |  | 1.051 | 0.3302 | 1.46E-03 |
| S8_121669693 | *a* | 0.3617 | 0.3298 | 0.0773 | 2.00E-05 |
| S8_125301167 | *a* | 1.2088 | 1.1437 | 0.0764 | 1.91E-50 |
| S8_150801746 | *a* | 1.5605 | 1.6276 | 0.0775 | 8.61E-97 |
| S9_105425957 | *a* | 0.6663 | 0.7395 | 0.0784 | 4.43E-21 |
| S9_109173783 | *a* | -0.7262 | -0.9375 | 0.0772 | 7.98E-34 |
| S9_146873044 | *a* | -0.7099 | -0.7734 | 0.0775 | 2.07E-23 |
|  | *de_3_* |  | -1.208 | 0.5508 | 2.83E-02 |
| S10_141070659 | *a* | 1.3206 | 1.227 | 0.0766 | 1.99E-57 |

Note: *a*: additive effect (the true effects in this simulation), *d*: dominance effect (falsely identified effect in this simulation), *de_1_*: environment 1 specific dominance effect (falsely identified effect in this simulation), *de_3_*: environment 3 specific dominance effect (falsely identified effect in this simulation). P-value: experimental-wise P value. For GWAS, we only report the effects with highly significant (*P*_EW_ < 1×10^-5^). Dominance and environment specific dominance effects were not highly significant in this simulation. None of the epistasis effects were significant in our simulation.
