# Supplementary material for: Dominance and Epistasis Interactions Revealed as Important Variants for Leaf Traits of Maize NAM Population
Source: Front Plant Sci. 2018 Jun 18;9:627. doi: 10.3389/fpls.2018.00627 (PMC6015889; doi:10.3389/fpls.2018.00627)
Supplement: Supplementary file 7 [file Table_7.DOC]

Table S7 **|** Comparison between the positions of identified loci and the reported loci by Tian et al. (2011) for upper leaf angle.

| **Identified SNPs** | | **Previously reported SNPs (Tian et al, 2011)** | | | **Previously reported QTL (Tian et al, 2011)** | |
| --- | --- | --- | --- | --- | --- | --- |
| SNP | BP | SNP | BP | cM | QTL | Support interval (cM) |
| S1_19944329 | 19944329 | PZE0126783951 | 26783951 | 44.116 | m33 | 41.7 - 48.5 |
| S1_35133062 | 35133062 | PZE0134648362 | 34648362 | 54.104 |  |  |
| S1_44956540 | 44956540 |  |  |  |  |  |
| S1_258657782 | 258657782 | PZE01247248533 | 247248533 | 145.351 | m122 | 146 - 150.1 |
| S1_273371612 | 273371612 | PZE01280647647 | 280647647 | 175.544 | m146 | 175 - 181.3 |
| S2_7180843 | 7180843 | PZE0205209256 | 5209256 | 15.424 | m189 | 10.2 - 14 |
| S2_62991412 | 62991412 |  |  |  |  |  |
| S3_162063504 | 162063504 | PZE03160701001 | 160701001 | 73.549 | m365 | 68.6 - 72 |
| S3_163994316 | 163994316 | PZE03164583838 | 164583838 | 75.78 |  |  |
| S3_171437505 | 171437505 | PZE03170869066 | 170869066 | 81.758 |  |  |
| S3_177144018 | 177144018 | PZE03175051481 | 175051481 | 85.335 | m381 | 84.2 - 85.7 |
| S3_177492233 | 177492233 | PZE03175740254 | 175740254 | 86.346 | m381 | 84.2 - 85.7 |
| S3_216647852 | 216647852 | PZE03215731660 | 215731660 | 129.642 | m426 | 140.6 - 151.2 |
| S4_185870953 | 185870953 | PZE04178879786 | 178879786 | 91.714 | m499 | 85.9 - 99.3 |
| S5_23504867 | 23504867 |  |  |  |  |  |
| S5_24377120 | 24377120 |  |  |  |  |  |
| S5_29918641 | 29918641 |  |  |  |  |  |
| S5_32090529 | 32090529 |  |  |  |  |  |
| S5_35629887 | 35629887 | PZE0535806901 | 35806901 | 58.15 | m585 | 57.2 - 58.6 |
| S5_63801506 | 63801506 | PZE0562647295 | 62647295 | 63.847 | m604 | 63.5 - 67.5 |
| S5_65225359 | 65225359 | PZE0564675306 | 64675306 | 64.216 | m604 | 63.5 - 67.5 |
| S5_84825303 | 84825303 | PZE0575406828 | 75406828 | 66.849 | m604 | 63.5 - 67.5 |
| S5_98223550 | 98223550 |  |  |  |  |  |
| S5_194091993 | 194091993 | PZE05199130596 | 199130596 | 106.732 | m659 | 102.6 - 107.6 |
| S5_201069419 | 201069419 | PZE05213249961 | 213249961 | 144.363 |  |  |
| S6_34565567 | 34565567 | PZE0628601861 | 28601861 | 7.982 |  |  |
| S6_60149057 | 60149057 | PZE0664380242 | 64380242 | 9.4 |  |  |
| S8_77253417 | 77253417 | PZE0891938969 | 91938969 | 56.181 | m877 | 55.9 - 56.1 |
| S8_78857312 | 78857312 | PZE0891938969 | 91938969 | 56.181 | m877 | 55.9 - 56.1 |
| S8_166675138 | 166675138 | PZE08166486198 | 166486198 | 105.543 |  |  |
| S9_28046935 | 28046935 | PZE0928951074 | 28951074 | 44.533 | m988 | 48.4 - 52.6 |
| S9_109173000 | 109173000 | PZE09105197539 | 105197539 | 52.208 | m988 | 48.4 - 52.6 |
| S9_142035936 | 142035936 | PZE09142010824 | 142010824 | 87.25 | m1017 | 87.4 - 92.4 |
| S9_146872957 | 146872957 | PZE09144169123 | 144169123 | 92.249 | m1017 | 87.4 - 92.4 |
| S10_59115236 | 59115236 |  |  |  |  |  |
| S10_60155825 | 60155825 |  |  |  |  |  |
| S10_144934798 | 144934798 | PZE10145190367 | 145190367 | 85.757 | m1098 | 76.7 - 86.4 |
